# Cyanobacteria and Eukaryotic Algae Use Different Chemical Variants of Vitamin B_12_

**DOI:** 10.1016/j.cub.2016.02.041

**Published:** 2016-04-25

**Authors:** Katherine Emma Helliwell, Andrew David Lawrence, Andre Holzer, Ulrich Johan Kudahl, Severin Sasso, Bernhard Kräutler, David John Scanlan, Martin James Warren, Alison Gail Smith

**Affiliations:** 1 Department of Plant Sciences, University of Cambridge, Cambridge CB2 3EA, UK; 2 School of Biosciences, University of Kent, Kent CT2 7NJ, UK; 3 Institute of Pharmacy and Molecular Biotechnology, Ruprecht-Karls University Heidelberg, Im Neuenheimer Feld 364, 69120 Heidelberg, Germany; 4 Institute of Organic Chemistry and Centre of Molecular Biosciences, University of Innsbruck, Innrain 80/82, 6020 Innsbruck, Austria; 5 School of Life Sciences, University of Warwick, Coventry CV4 7AL, UK

**Keywords:** vitamin B_12_, cyanobacteria, pseudocobalamin, algae, competition, nutrient cycling, phytoplankton

## Abstract

Eukaryotic microalgae and prokaryotic cyanobacteria are the major components of the phytoplankton. Determining factors that govern growth of these primary producers, and how they interact, is therefore essential to understanding aquatic ecosystem productivity. Over half of microalgal species representing marine and freshwater habitats require for growth the corrinoid cofactor B_12_, which is synthesized de novo only by certain prokaryotes, including the majority of cyanobacteria. There are several chemical variants of B_12_, which are not necessarily functionally interchangeable. Cobalamin, the form bioavailable to humans, has as its lower axial ligand 5,6-dimethylbenzimidazole (DMB). Here, we show that the abundant marine cyanobacterium *Synechococcus* synthesizes only pseudocobalamin, in which the lower axial ligand is adenine. Moreover, bioinformatic searches of over 100 sequenced cyanobacterial genomes for B_12_ biosynthesis genes, including those involved in nucleotide loop assembly, suggest this is the form synthesized by cyanobacteria more broadly. We further demonstrate that pseudocobalamin is several orders of magnitude less bioavailable than cobalamin to several B_12_-dependent microalgae representing diverse lineages. This indicates that the two major phytoplankton groups use a different B_12_ currency. However, in an intriguing twist, some microalgal species can use pseudocobalamin if DMB is provided, suggesting that they are able to remodel the cofactor, whereas *Synechococcus* cannot. This species-specific attribute implicates algal remodelers as novel and keystone players of the B_12_ cycle, transforming our perception of the dynamics and complexity of the flux of this nutrient in aquatic ecosystems.

## Highlights

• Dominant marine cyanobacteria synthesize only pseudocobalamin
• Pseudocobalamin is orders of magnitude less bioavailable to eukaryotic algae
• Certain algae can remodel pseudocobalamin to a bioavailable form
• This implies a complex B_12_ cycle between microbes in the photic zone

## Graphical Abstract

**Figure:**
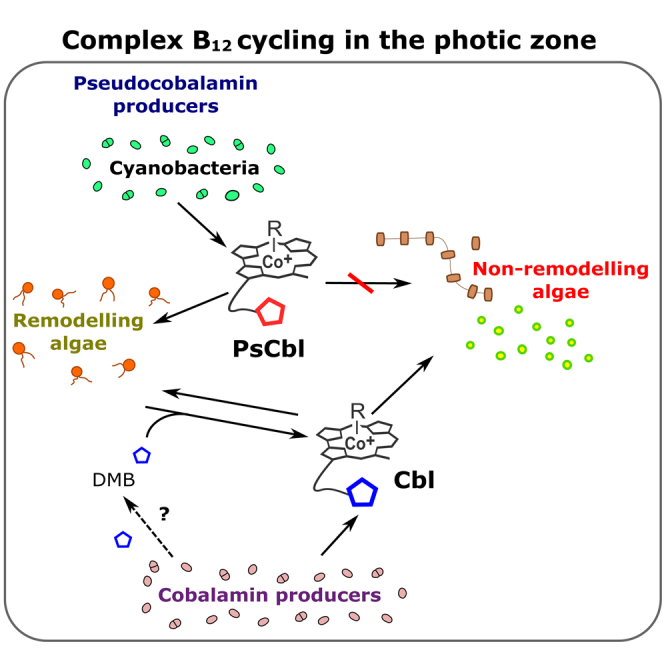

## Teaser

Helliwell et al. demonstrate that the two major groups of photosynthetic microbes in the photic zone, cyanobacteria and microalgae, use different forms of vitamin B_12_. These findings challenge the oversimplified assumption of a linear flux of B_12_ from producers to consumers, in favor of a more complex network of B_12_ production, uptake, and cycling.

## Introduction

Eukaryotic microalgae are photosynthetic microbes estimated to be responsible for up to 50% of global carbon fixation [1]. Elucidation of factors that control algal community structure and dynamics is thus fundamental to understanding the global cycling of carbon. Nutrients such as iron, nitrogen, and phosphorus clearly play an important role [2], but many microalgae also require the vitamins B_1_ (thiamine), B_7_ (biotin), or B_12_ for growth [3]. B_12_ is required as a cofactor for methionine synthase (METH; EC 2.1.1.13) activity, a key enzyme of cellular one-carbon (C1) metabolism important for production of the universal methyl donor S-adenosylmethionine (SAM), and for folate cycling necessary for DNA synthesis [4]. Those algae that do not need a supply of B_12_ cannot synthesize the vitamin; rather, they possess an alternative form of methionine synthase (METE; EC 2.1.1.14) that can catalyze the same reaction in a B_12_-independent fashion [5, 6].

Measurement of B_12_ levels in the water column have indicated concentrations of ∼10 pM in freshwater ecosystems [7] and are often below the threshold of detection in certain marine habitats, including large areas of the northeast Pacific margin [8]. The scarcity of this micronutrient is therefore thought to limit phytoplankton abundance [8], so competition for B_12_ among those organisms that require/use it is likely. Indeed, field-enrichment experiments found that, whereas N addition stimulated all microbial growth, there was a specific growth enhancement of phytoplankton >5 μm (i.e., the larger eukaryote fraction) with B_12_ supplementation [9]. However, several recent studies have demonstrated that heterotrophic bacteria can satisfy microalgal requirements for B_12_ via mutualistic interactions (e.g., [5, 10]).

**Figure 1:**
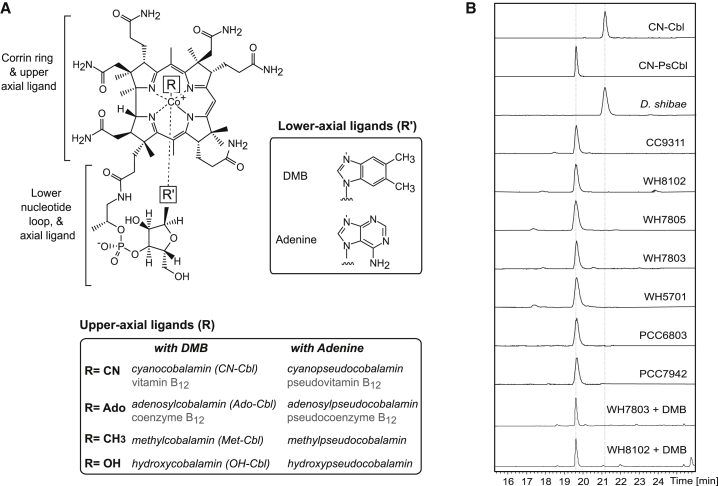
*Synechococcus* Strains Synthesize Pseudocobalamin Rather Than Cobalamin (A) Structural variants of B_12._ (B) HPLC-MS extracted ion chromatograms for m/z 1355.5 cyanocobalamin and m/z 1344.5 cyanopseudocobalamin (see also Figure S1). The lower tracks display the chromatograms for cell-free extracts derived from cultures of cyanobacterial species from Figure S2. Experiments were carried out in triplicate, and one replicate representative of each strain is shown.

Provision from prokaryotes is particularly pertinent because the biosynthetic pathway of this cofactor is confined to certain archaea and bacteria. B_12_ is an umbrella term that refers to cobalt-containing corrinoids (ring-contracted tetrapyrroles), which have upper and lower axial ligands to the cobalt ion (Figure 1A). The nature of these ligands varies, leading to diversity in the structural forms of B_12_. Methylcobalamin, where the upper axial ligand is a methyl group, is involved in methyl-transfer reactions, whereas adenosylcobalamin (coenzyme B_12_) is used for radical-based rearrangements and reductions [11]. The identity of the base found as the lower axial ligand, bound via a nucleotide loop, may vary too. In cobalamin, the base is 5, 6-dimethylbenzimidazole (DMB). Many bacteria, including methanogens and anaerobes such as *Clostridium* species, synthesize a B_12_ vitamer known as pseudocobalamin [12, 13], which differs from cobalamin in that DMB is replaced by adenine. Intrinsic factor, the mammalian B_12_-binding protein important in uptake from the gut, has a lower affinity for pseudocobalamin than cobalamin [14]. Pseudocobalamin is therefore considered not “bioavailable” to humans, and the efficacy of vitamin supplements produced from cyanobacteria such as *Spirulina* and *Aphanizomenon*, which also contain pseudocobalamin, has been questioned [15, 16].

Cyanobacteria are the numerically dominant photosynthetic microbes in the marine environment [17]. Two cyanobacterial strains, *Crocosphaera watsonii* WH8501 and *Synechococcus* sp. WH7803, were reported to release B_12_ into the media at rates exceeding those estimated for the heterotrophic bacterium *Halomonas*, suggesting that cyanobacteria might be the major source of B_12_ for marine algae [18]. However, indications from the early literature suggest consideration of algal specificity toward different B_12_-like factors may be pertinent [19]. Here, we investigate corrinoids in several strains of *Synechococcus*, an abundant and ubiquitous marine cyanobacterium [17, 20]; the nature of their axial ligands; and their ability to support growth of eukaryotic primary producers.

## Results

### *Synechococcus* Species Make Only Pseudocobalamin

The biosynthesis of the corrinoid ring of B_12_ from the common tetrapyrrole progenitor uroporphyrinogen III requires at least 20 enzymatic steps, and several routes are known [21]. In a preliminary investigation, Sañudo-Wilhelmy et al. [22] searched for the presence of B_12_-biosynthesis genes in ∼40 marine cyanobacteria with sequenced genomes. They found that all but one species had putative homologs for at least 11 of these genes and so concluded that they were capable of making B_12_. However, they did not investigate the genes involved in synthesis of the lower axial ligand and so could not conclude whether the cyanobacteria studied produced cobalamin or pseudocobalamin. To refine the analysis of B_12_-biosynthesis genes, we searched 123 sequenced cyanobacterial genomes for all 20 genes of the corrinoid pathway. All but six species contained at least 15/20 of these genes and were predicted to be B_12_ producers (Data S1; Supplemental Experimental Procedures). Additionally, we searched for genes involved in DMB biosynthesis, for which two routes are currently known [23, 24]. The BluB enzyme, first characterized in *Sinorhizobium meliloti* (Rhizobia) makes DMB from riboflavin under aerobic conditions [23]. Using this sequence as a query, no hits were found in 118 cyanobacterial genomes including *C. watsonii* WH8501 and *Synechococcus* sp. WH7803. For five species (including three from the *Fischerella* genus), hits for BluB were obtained: two were annotated as cob(II)yrinic acid *a,c*-diamide reductase (CobR), which is an enzyme of an earlier stage of B_12_ biosynthesis, whereas the others were unknown. In contrast, BluB homologs were found in 80% of sequenced rhizobia (227/284 genomes; Data S1C) and 60% of Rhodobacterales species (77/128; Data S1D) including *Mesorhizobium loti*, *Sinorhizobium meliloti*, *Rhizobium leguminosarum*, and the marine bacterium *Dinoroseobacter shibae*, all of which can support algal B_12_-auxotrophic growth [10, 25]. More recently, a second route for DMB biosynthesis was identified in the obligate anaerobic bacterium *Eubacterium limosum* [24], and enzymes encoded by the *bzaABCDE* operon were shown to direct DMB production via an oxygen-sensitive reaction from the purine precursor 5-aminoimidazole ribotide (AIR) [24]. We found that none of the cyanobacterial genomes encoded the full *bzaABCDE* operon (Data S1B). Moreover, CobT, which is required for DMB activation, is absent from all but two cyanobacterial genomes. Taken together, these searches suggest that the vast majority of cyanobacteria cannot make DMB.

To validate the observations from the bioinformatics analysis, we wanted to assess directly what corrinoids are synthesized by cyanobacteria so investigated the B_12_ content of strains of marine *Synechococcus*, because this is an ancient and ecologically abundant lineage [20] with a mean global abundance of 7.0 ± 0.3 × 10^26^ cells y^−1^, high-biomass-specific CO_2_ fixation rates [17, 26] and axenic strains are available. Corrinoids can be extracted from cells as their cyano-derivatives and then analyzed by high-performance liquid chromatography (HPLC)-mass spectrometry (LC-MS). First, using purified cyanocobalamin (obtained commercially) and cyanopseudocobalamin, prepared from *Propionibacterium acidi-propionici* [12], we were able to distinguish the two variants of B_12_ by their different retention times on the LC (Figure 1B) and different mass (Figures S1A and S1B). Derivatized cell lysate obtained from axenic cultures of the heterotrophic marine bacterium *D. shibae* DFL12T contained only cyanocobalamin. We next tested five members of the *Synechococcus* lineage representing different clades and habitats (highlighted in red in Figure S2): coastal strain CC9311 (sub-cluster [SC] 5.1A; clade I); oligotroph WH8102 (SC5.1A; clade III); WH7803 and WH7805 (SC5.1B; clades V and VI, respectively) which are widely distributed in various oceanic waters; and the estuarine strain WH5701 (SC5.2). A single peak was observable in these samples at a retention time consistent with the pseudocobalamin standard (Figure 1B), and its identity was confirmed by MS (Figures S1D–S1H). To facilitate subsequent physiological work, we also tested two model freshwater cyanobacterial species *Synechocystis* sp. PCC6803 and *Synechococcus elongatus* PCC7942, because these species grow quickly and easily in the laboratory. Again, cell lysates from these strains contained only pseudocobalamin (Figures 1B, S1I, and S1J). Together, these data demonstrate that the cyanobacterial species sampled here make only pseudocobalamin in axenic laboratory culture conditions.

However, some B_12_-synthesizing bacteria can modify endogenous B_12_ forms with an alternative base [27]. For instance, although *Salmonella enterica* cannot make DMB, it can import it and then make cobalamin instead of pseudocobalamin [27]. To investigate whether *Synechococcus* can perform this so-called “guided biosynthesis,” we grew strains WH8102 and WH7803 (representing SC5.1A and 5.1B) in the presence of pseudocobalamin and 1 μM DMB, but only pseudocobalamin was detected (Figures 1B, S1K, and S1L). We conclude therefore that *Synechococcus* cannot replace the adenine base with DMB to make cobalamin.

### Pseudocobalamin Is Orders of Magnitude Less Bioavailable to Eukaryotic Algae

**Figure 2:**
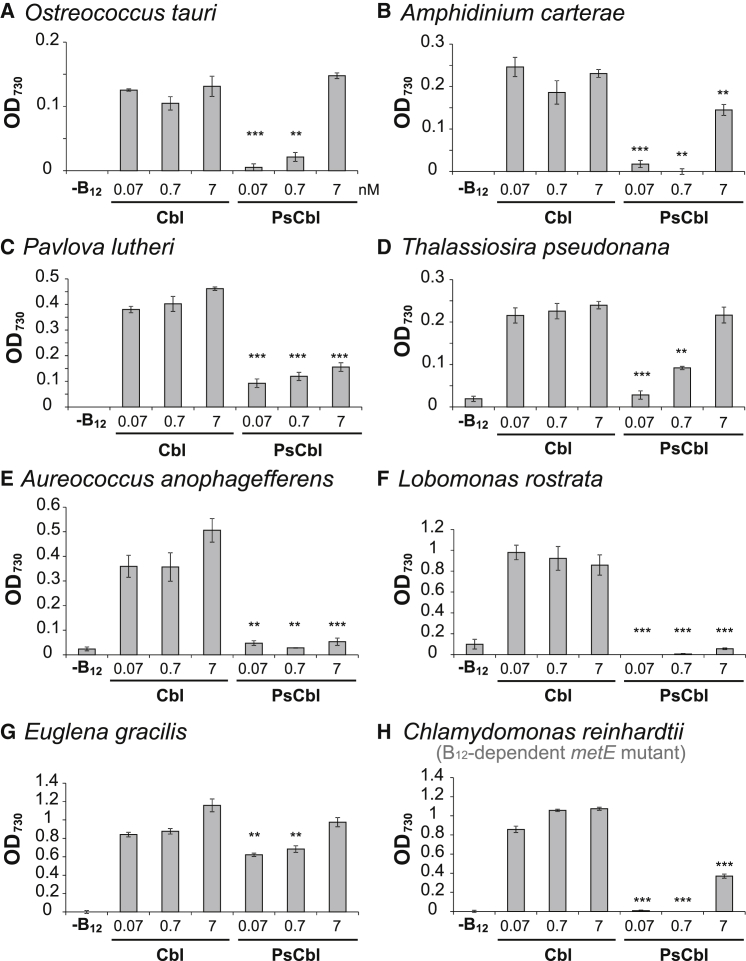
Pseudocobalamin Poorly Supports Growth of B_12_-Dependent Eukaryotic Algae (A–H) Growth yield (OD_730_) of algae in liquid medium supplemented with or without cyanocobalamin or cyanopseudocobalamin (at 0.07, 0.7, or 7 nM) in batch culture after at least two transfers (until the cells had died in the −B_12_ treatment). (A) *O. tauri*, (B) *A. carterae*, (C) *P. lutheri*, (D) *T. pseudonana*, (E) *A. anophagefferens*, (F) *L. rostrata*, (G) *E. gracilis*, and (H) *C. reinhardtii* B_12_-dependent *metE* mutants are shown [29]. Cbl, cyanocobalamin; PsCbl, cyanopseudocobalamin. ^∗^p ≤ 0.05; ^∗∗^p ≤ 0.01; ^∗∗∗^p ≤ 0.001 compared with the equivalent concentration of Cbl (two-tailed Student’s t test; mean ± SEM; n = 3). See also Figures S3 and S4.

We next tested whether cyanobacterially derived B_12_ could be utilized by eukaryotic algae. Cell-free extracts of *S. elongatus* PCC7942 (pseudocobalamin producer) were unable to rescue growth of the B_12_-dependent freshwater alga *Lobomonas rostrata*, whereas there is clear growth with the addition of extracts of three rhizobial bacteria (Figure S3), which all encode BluB [28]. It is conceivable that the growth is due to other compounds in the crude lysate, so this initial experiment was extended using the purified compounds, cyanocobalamin and cyanopseudocobalamin. Equivalent concentrations of each B_12_ variant were added to axenic cultures of B_12_-dependent microalgae from different algal lineages: marine species *Ostreococcus tauri* (Chlorophyta, Mamiellophyceae); *Amphidinium carterae* (Alveolata, Dinoflagellate); *Pavlova lutheri* (Haptophyta, Prymnesiophyceae); *Thalassiosira pseudonana* (Heterokontophyta, Coscinodiscophyceae); *Aureococcus anophagefferens* (Heterokontophyta, Pelagophyceae); and the freshwater species *Euglena gracilis* (Excavata, Euglenozoa) and *L. rostrata* (Chlorophyta, Volvocales). We also tested a B_12_-dependent *metE* mutant of *Chlamydomonas reinhardtii* (Chlorophyta, Volvocales) [29]. When pseudocobalamin was supplied at a concentration of 0.07 or 0.7 nM, we observed little or no growth in any of the marine species, nor with the *C. reinhardtii metE* mutant or *L. rostrata*. This is in contrast to cobalamin, which supported growth of all algal cultures at equivalent concentrations (Figure 2; Student’s t test; p < 0.05; n = 3). For *O. tauri*, *A. carterae*, and *T. pseudonana*, and to a lesser extent the *C. reinhardtii metE* mutant, provision of pseudocobalamin at 7 nM (∼10 μg/l) supported growth to a similar extent as cobalamin (Figures 2A, 2B, 2D, and 2H), although this amount is significantly higher than found in natural ecosystems (with reported concentrations ranging from below the detection threshold to 0.03 nM across large areas of the northeast Pacific margin, for instance) [7, 8]. One way to compare the efficacy of the different B_12_ variants is to carry out dose-response experiments, which enable determination of an EC_50_ (that is the effective concentration required to support half-maximal biomass accumulation) and also provide an indication of the minimum amount needed to observe any growth, and so we carried these out with the *C. reinhardtii metE* mutant. Figure S4 shows that the EC_50_ was ∼0.07 nM for cobalamin, compared to ∼7 nM (∼100-fold higher) for pseudocobalamin. In addition, it is clear that even the highest concentration of pseudocobalamin used (40 nM) is not saturating, whereas 0.2 nM cobalamin supports maximum growth. For *E. gracilis*, some growth was observed even at the lowest pseudocobalamin concentration, but it was still significantly lower than with cobalamin (Figure 2G). Thus, pseudocobalamin is orders of magnitude less bioavailable to eukaryotic algae. It is notable to mention that *E. gracilis* has also been demonstrated to encode a B_12_-dependent (type II) ribonucleotide reductase [30], which could account for the growth response to pseudocobalamin observed in this alga.

**Figure 3:**
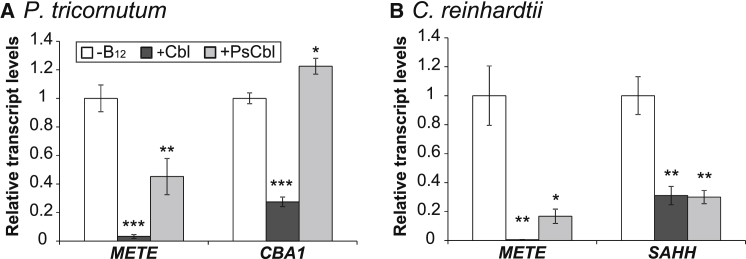
Pseudocobalamin Affects Expression of B_12_-Responsive Genes in *P. tricornutum* and *C. reinhardtii* (A and B) qRT-PCR analysis of (A) *METE* and *CBA1* expression in *P. tricornutum* and (B) *METE* and *SAHH* expression in *C. reinhardtii,* without B_12_ or with 0.7 nM Cbl/PsCbl. Expression was normalized using three housekeeping genes: *Histone H4*; 30S, ribosomal protein S1, *RPS*; and TATA box-binding protein, *TBP* for *P. tricornutum* and receptor of activated *protein* kinase C 1, *RACK 1*; Actin, *ACT*; ubiquitin, *UBQ* for *C. reinhardtii*. ^∗^p ≤ 0.05; ^∗∗^p ≤ 0.01; ^∗∗∗^p ≤ 0.001 compared to the −B_12_ treatment (two-tailed Student’s t test; mean ± SEM; n = 3). See also Figure S5.

We reasoned that the reduced ability of pseudocobalamin to support growth of algal B_12_-auxotrophs may be either because the molecule cannot be used as a cofactor or because it does not get transported into algal cells. To investigate the latter possibility, we took advantage of the presence of B_12_-responsive genes previously identified in the marine diatom *Phaeodactylum tricornutum* and *C. reinhardtii* [31, 32]. These algae do not need B_12_ for growth but will uptake and use it if it is available [5, 6]. Several genes in these algae are responsive to B_12_: *METE* (in *P. tricornutum* and *C. reinhardtii*) [6, 31, 32]; *CBA1*, encoding a novel cobalamin acquisition protein (in *P. tricornutum* only) [31]; and S-adenosylhomocysteine hydrolase, *SAHH* (in *C. reinhardtii* only) [32]. Using qRT-PCR, we analyzed their expression in cells grown in the presence of 0.7 nM cobalamin or pseudocobalamin. For *P. tricornutum*, both forms of B_12_ resulted in downregulation of *METE,* but the effect was less pronounced with pseudocobalamin compared to that with cobalamin (Student’s t test; p < 0.001; n = 3; Figure 3A). As previously demonstrated, cobalamin suppressed *CBA1* [31], but this gene was significantly upregulated by pseudocobalamin (Student’s t test; p < 0.05; n = 3; Figure 3A). In *C. reinhardtii*, both *METE* (Student’s t test; p < 0.05; n = 3) and *SAHH* (Student’s t test; p < 0.01; n = 3) were downregulated relative to the no supplementation control (Figure 3B) with both forms of B_12_. Subsequent western blot analysis using polyclonal antibodies against *C. reinhardtii* METE protein [32] demonstrated a modest reduction of METE abundance when cells were grown with pseudocobalamin, although not to the same extent as cobalamin (Figure S5). Nonetheless, the effect of pseudocobalamin on the expression of these four B_12_-responsive genes indicates that the molecule can enter both *C. reinhardtii* and *P. tricornutum* cells.

### Certain Algae Are Capable of Remodeling Pseudocobalamin

**Figure 4:**
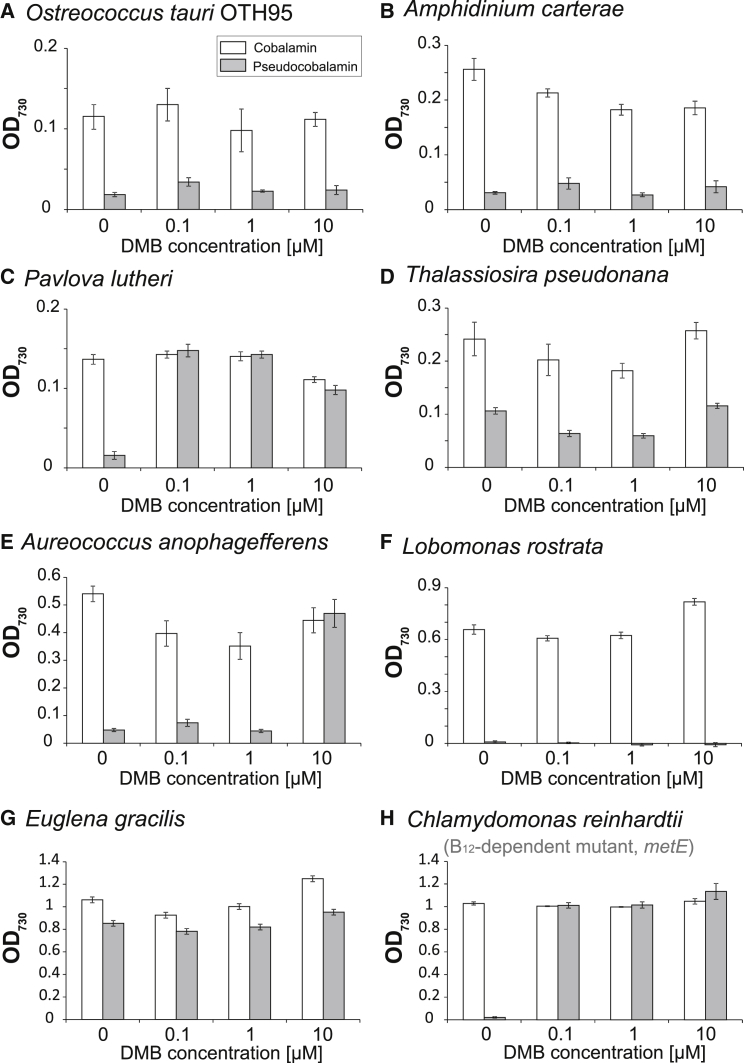
Provision of Lower Ligand Substrate DMB Together with Pseudocobalamin Can Support Growth of Certain B_12_-Dependent Algae (A–H) Species were grown in liquid medium (Table S2) without or with 0.7 nM cobalamin (open bars) or 0.7 nM pseudocobalamin (shaded bars) in the presence of different DMB concentrations in batch culture over several transfers or until the cells had died in the −B_12_ treatment. (A) *O. tauri* (OTH95), (B) *A. carteri*, (C) *P. lutheri*, (D) *T. pseudonana*, (E) *A. anophagefferens*, (F) *L. rostrata*, (G) *E. gracilis*, and (H) *C. reinhardtii,* B_12_-dependent evolved (*metE*) mutant lines are shown [29]. Optical density (OD_730_) was used to quantify growth (mean ± SEM; n = 3).

**Figure 5:**
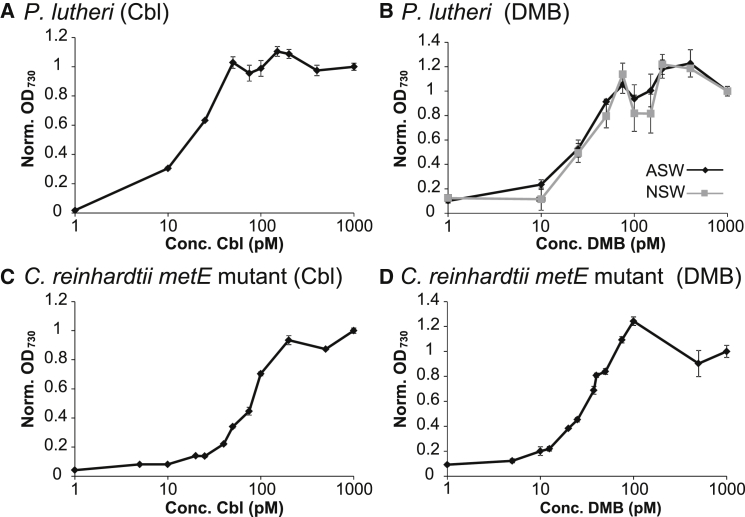
Certain Algae Can Remodel the Lower Axial Ligand of Pseudocobalamin with Exogenously Supplied DMB (A and B) Relative growth yield of (A) *P. lutheri* cells supplemented with different concentrations of cobalamin after 19 days (values of OD_730_ were normalized as a proportion of growth at 1,000 pM B_12_) or (B) *P. lutheri* cells supplemented with different concentrations of DMB in the presence of 0.7 nM pseudocobalamin (after 19 days) in artificial seawater or natural filtered seawater (values of OD_730_ normalized to growth at 1,000 pM DMB). (C and D) Equivalent experiments with *C. reinhardtii*, evolved B_12_-dependent *metE* mutant [29] are displayed in (C) and (D) after 96 hr growth (mean ± SEM; n = 3).

**Figure 6:**
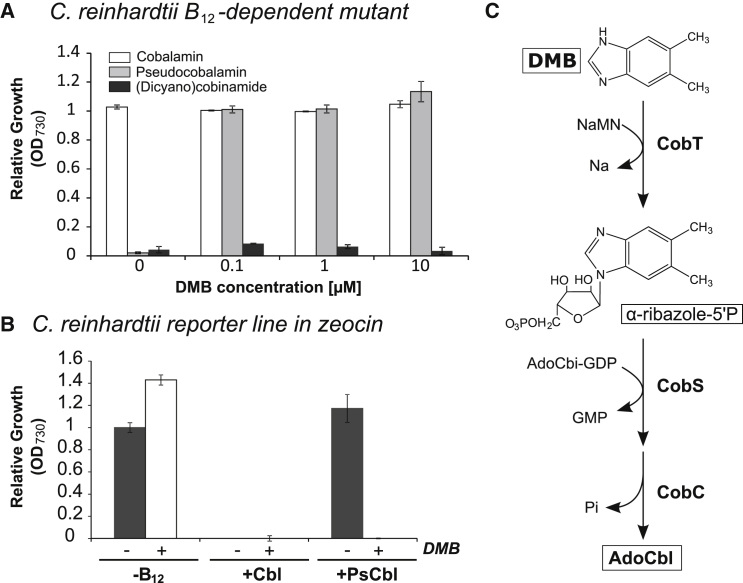
Further Characterization of the Remodeling Phenotype (A) Relative growth yield (OD_730_) of *C. reinhardtii* B_12_-dependent mutant grown with DMB alongside (dicyano)cobinamide, a B_12_ precursor that lacks the DMB ribonucleotide tail. Cells were grown in liquid medium (Table S2) with 0.7 nM cobalamin (open bars), 0.7 nM pseudocobalamin (gray bars), or 0.7 nM (dicyano)cobinamide (black bars) in the presence of different DMB concentrations. (B) Relative growth yield (OD_730_) of *C. reinhardtii* reporter line containing a Zeocin resistance gene controlled by the *METE* promoter [32] after 13 days in the presence (white bar) or absence (black bar) of DMB (1 μM) and 20 μg/ml Zeocin without or with 0.7 nM cobalamin or pseudocobalamin. Values of OD_730_ were normalized as a proportion of growth with no B_12_ (mean ± SEM; n = 3). (C) The pathway for the activation of DMB and nucleotide loop assembly in *S. enterica* (adapted from [27]). CobT catalyzes the attachment of a phosphoribose moiety derived from nicotinate mononucleotide to form α-ribazole phosphate. CobS and CobC catalyze the attachment of the activated base to the cobamide precursor (GDP-cobinamide).

By analogy with the guided biosynthesis described earlier, some bacteria that cannot synthesize B_12_ de novo can modify imported forms with an alternative base, via “corrinoid re-modeling” [33, 34]. To investigate this possibility in algae, we grew B_12_-requiring species in the presence of pseudocobalamin and a range of DMB concentrations. For most, growth was not restored by DMB supplementation (Figures 4A, 4B, and 4D–4G). However, for *P. lutheri* and the *C. reinhardtii metE* mutant, addition of DMB alongside pseudocobalamin rescued growth to the same extent as cobalamin (Figures 4C and 4H). A dose-response experiment with *P. lutheri* established an EC_50_ value of ∼18 pM for cobalamin (Figure 5A). A similar experiment with a fixed concentration (0.7 nM) of pseudocobalamin but varying the amount of DMB revealed a similar EC_50_ (∼23 pM; Figure 5B). Interestingly, an equivalent dose-response curve (and EC_50_ value: ∼26 pM) was observed when cells were grown in medium made using natural filtered seawater rather than artificial sea salts. Thus, the level of DMB in the natural filtered seawater is not sufficient to allow remodeling; otherwise, the dose-response curve would be shifted to the left. That comparable levels of B_12_ and DMB (at a fixed level of pseudocobalamin) are able to rescue B_12_-dependent growth implies that *P. lutheri* is remodeling pseudocobalamin with DMB to generate cobalamin. Dosage experiments with the *C. reinhardtii* B_12_-dependent *metE* mutant also identified similar EC_50_ values of ∼28 pM and ∼70 pM for cobalamin and DMB, respectively (Figures 5C and 5D). In this case, the EC_50_ value for DMB was slightly higher than cobalamin. We also tested whether *C. reinhardtii* is capable of de novo lower-loop synthesis and grew the *C. reinhardtii* B_12_-dependent *metE* mutant with DMB, alongside (dicyano)cobinamide, a B_12_ precursor that lacks the DMB ribonucleotide tail, but no restoration of growth was observed (Figure 6A).

Our data indicate that, of eight diverse algal species studied, six do not appear to be able to use exogenous DMB. Nevertheless, the observation that growth of the *C. reinhardtii* B_12_-dependent *metE* mutant (alongside that of *P. lutheri*) with pseudocobalamin is restored by DMB provision suggests that these algae are able to chemically modify pseudocobalamin to a form that can support B_12_-auxotrophic growth. To test more directly whether pseudocobalamin is being remodeled, we grew samples of *C. reinhardtii* in the presence of (1) cobalamin, (2) pseudocobalamin (1 nM), and (3) pseudocobalamin (1 nM) + DMB (1 μM) and prepared cell lysate for LC-MS analysis. However, we could not detect any form of B_12_ from lysed cells. We infer from this that intracellular B_12_ levels are extremely low, i.e., the quantity from ∼1 × 10^10^ cells is below the threshold detection of the LC-MS (which in our system is ∼1 × 10^12^ molecules). Without a clear idea of what order of magnitude more biomass would be required and constrained by the limitations of scale, we turned to alternative means of characterizing remodeling activity and investigated the effect of DMB + pseudocobalamin on gene expression in *C. reinhardtii.* Previously, we had generated several transgenic lines of *C. reinhardtii* expressing the B_12_-responsive element of the *METE* gene fused to the *BLE* gene, which confers resistance to the antibiotic Zeocin [32]. This reporter gene construct enables rapid and easy measurement of B_12_-responsive gene expression, whereby growth with cobalamin represses expression of *BLE* so that cells die in the presence of Zeocin (Figure 6B). In contrast, pseudocobalamin alone had little effect, but the inclusion of DMB impaired growth to the same extent as cobalamin, demonstrating that *C. reinhardtii* converts DMB and pseudocobalamin into a form that is able to repress the *METE* promoter.

The pathway for pseudocobalamin remodeling has been investigated previously in the purple bacterium *Rhodobacter sphaeroides*, and cobinamide amidohydrolase (CbiZ) and cobinamide-phosphate synthase (CbiB) have been implicated in this process [34]. We could not identify *cbiZ* or *cbiB* in any of the algal genomes we analyzed. We therefore searched for proteins shown to be involved in lower-loop assembly and activation [34] in *S. enterica* (CobT, CobS, and CobC) [27], where mutants of CobT are unable to incorporate exogenous DMB (Figure 6C). We identified genes encoding all three of these proteins in *C. reinhardtii*, which exhibits the remodeling phenotype (Table S1; Figure 5). In contrast, BLASTP searches of the genomes of *O. tauri* and *T. pseudonana*, species that do not appear to remodel, were negative for CobT and CobS. Although a hit for CobC was identified in *T. pseudonana*, it should be noted that CobC catalyzes a dephosphorylation step [21, 27], and therefore BLAST searches may retrieve genes encoding unrelated phosphatases. Interestingly, we identified hits for CobT and CobC, but not CobS, in *A. anophagefferens*, which can use pseudocobalamin with DMB, but only with very high levels of the latter (10 μM; Figure 4E). Transcript sequences for *P. lutheri* are available via the Marine Microbial Eukaryote Transcriptome Sequencing Project (MMETSP), a database of 396 unique strains representing ecologically significant and taxonomically diverse marine microbial eukaryotes [35]. This alga, which can use pseudocobalamin alongside DMB, also expresses *COBT*, *COBS*, and *COBC* (Data S2A). Thus, the presence of these novel proteins correlates with the ability to remodel pseudocobalamin, implicating them in B_12_ metabolism. We also identified another 46 candidate remodelers in the MMETSP (Data S2A), including several that encoded *METE*, and so are like *C. reinhardtii* in being independent of a source of B_12_ for growth. In total, the potential remodelers represented ∼11% of unique strains and included representatives of the higher class levels Alveolata, Stramenopila, Hacrobia, and Viridiplantae (Data S2B). Incidentally, none of the sequenced *Synechococcus* genomes encode CobT (Data S1), which might explain why *Synechococcus* strains cannot remodel pseudocobalamin to cobalamin in the presence of DMB (Figure 1B).

## Discussion

Eukaryotic microalgae and cyanobacteria are the major components of the phytoplankton in marine and freshwater systems. Because they both inhabit the photic zone, they will compete for resources including light and limiting nutrients such as nitrogen and Fe. We have demonstrated that, in contrast to heterotrophic bacteria such as *D. shibae* (and certain rhizobial bacteria) [28] that make cobalamin, members of the ubiquitous marine *Synechococcus* genus synthesize only pseudocobalamin, in which the lower base is adenine instead of DMB (Figure 1). Moreover, a survey of diverse cyanobacterial genomes, encompassing marine and freshwater species, showed the vast majority do not encode *bluB* or the *bzaABCDE* operon (Data S1) [23, 24]. This strongly suggests that pseudocobalamin is the major form of B_12_ synthesized by most if not all cyanobacteria.

We found that pseudocobalamin is considerably less bioavailable than cobalamin to several B_12_-dependent algae (Figure 2). This reduced bioavailability suggests these organisms are compromised in their ability to acquire or use pseudocobalamin as a cofactor. Human intrinsic factor, part of the B_12_ uptake system in the gut, exhibits a 500-fold-lower binding affinity for pseudocobalamin [14], thus reducing the bioavailability of the compound to humans. In algae, currently only one protein has been implicated in B_12_ uptake [31] (CBA1), although the precise molecular mechanism and role of CBA1 in B_12_ binding are not fully understood. Nevertheless, the ability of pseudocobalamin to affect the expression of algal B_12_-responsive genes (Figure 3) and protein levels (Figure S5) suggests this compound can enter algal cells, albeit that it has the opposite effect on *CBA1* to cobalamin, suggesting that the cells are experiencing cobalamin deficiency. Transport of pseudocobalamin into the cell is also indicated by our observed remodeling of pseudocobalamin in *C. reinhardtii* and *P. lutheri* following DMB addition (Figures 4 and 5). The identification of genes encoding enzymes of lower ligand activation (COBT) and nucleotide-loop assembly (COBS) [27] in these algae (Table S1) provides a likely mechanism for corrinoid remodeling. We found no evidence of secretory peptide signals in *C. reinhardtii* COBT or COBS using the green algal subcellular localization tool “PredAlgo” [36], implying that remodeling takes place within the cell and providing further support for the ability of pseudocobalamin to be taken up. Whether these genes have been acquired through lateral gene transfer from a bacterial source, which is thought to be the case for *E. coli* [37], remains unknown. However, of the non-algal sequences retrieved via a BLAST search of the NCBI non-redundant database with the *C. reinhardtii COBT/COBS*, top hits were derived from the amphipod-associated protist species *Aphanomyces astaci* (4e−57) and *Sphaeroforma arctica* (7e−77), respectively. A broader phylogenetic analysis of *COB* genes will be integral to further understanding of what, at a first glance, appears to be an intriguing evolutionary history. Because algae rely on B_12_ for METH [5], the function of pseudocobalamin as a cofactor for this enzyme is also an important question. Structural data available for the B_12_-binding pocket and the active site of METH [38, 39] implicate several amino acids in B_12_ binding, with the DMB “tail” buried within a cleft of the active site [40]. Because pseudocobalamin contains an alternative lower base to B_12_, it seems plausible that algal METH proteins may have reduced binding affinity for pseudocobalamin.

The combination of DMB with pseudocobalamin improves the bioavailability to certain algae. We infer from this that these remodeling algae are able to generate cobalamin from pseudocobalamin + DMB, although we were unable to measure detectable levels of any form of B_12_ in *C. reinhardtii* cells grown under these conditions. It is possible therefore that another form of the vitamin is being generated, though we deem this unlikely. In any case, our results highlight the importance of considering environmental concentrations of DMB. A bioassay to measure free DMB concentrations was recently reported [41]. Analysis of samples derived from host-associated (termite/rumen) and natural environmental samples (*Eucalyptus* grove soil/creek water) determined concentrations in the picomolar and sub-picomolar range. Our observation that *P. lutheri* (whose growth can be supported by pseudocobalamin + DMB) could not grow in natural seawater from the English Channel supplemented with pseudocobalamin (Figure 5B) suggests DMB levels were not sufficient in these coastal waters to support remodeling. Nevertheless, further work is required to quantify DMB in marine (and freshwater) environments more broadly. In a similar vein, a recent field study by Heal et al. [42] quantified the relative abundance of four upper axial variants of B_12_ (CN, Me, Ado, and OH) in estuarine waters of the Puget Sound, but levels of lower axial ligand variants are unknown.

**Figure 7:**
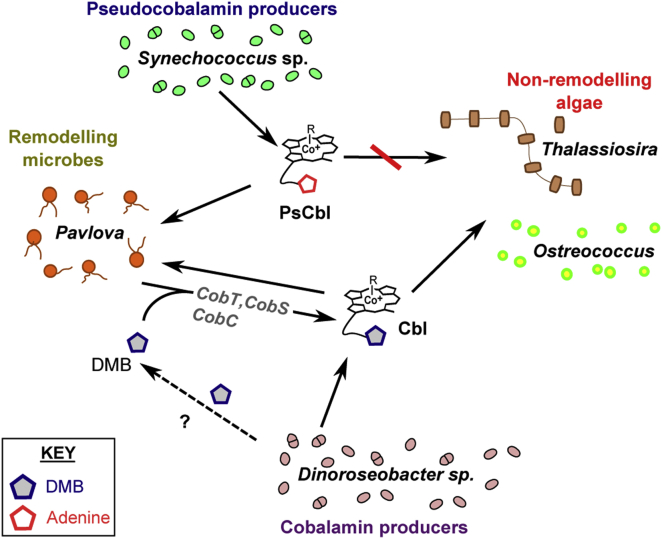
Complex B_12_ Cycling in a Hypothetical Marine Microbial Community Cobalamin produced by heterotrophic bacteria such as *Dinoroseobacter* sp. is directly usable by algal B_12_ auxotrophs representing major marine taxa, whereas cyanobacterially derived pseudocobalamin is not. However, those algae like *P. lutheri* capable of remodelling can access this essential cofactor if DMB is also present.

Representatives of all major algal lineages require B_12_, yet its biosynthesis is limited to a subset of prokaryotes. As such, the flux of B_12_ between microbes is integral to the growth of auxotrophic species. Our results imply heterotrophic bacteria are likely to be a more important source of B_12_ for eukaryotic algae than cyanobacteria. An increasing body of evidence provided by this study and others [33, 34, 43, 44] suggests the relationship between requirers and providers has become blurred by the existence of scavengers and remodelers. That different B_12_ forms are not functionally equivalent between organisms means that biochemical transformations between vitamer classes are essential for this micronutrient to reach different members of the community. This complicates our current view of B_12_ cycling in aquatic environments (Figure 7). Whether cyanobacteria synthesize a currency of B_12_ that is inaccessible to competing eukaryotic microbes as a strategy to exclude competitors remains unknown. Nevertheless, the observation that certain algae possess a counter-mechanism to convert pseudocobalamin to a bioavailable form suggests the selective pressure to devise and refine strategies of B_12_ acquisition/utilization in order to enhance accessibility to this limiting micronutrient is strong. In any case, the importance of B_12_ and its derivatives in structuring microbial communities in aquatic ecosystems may have been previously underestimated.

## Experimental Procedures

### Bioinformatics Approaches

A full description of sequence similarity search parameters is provided in the Supplemental Experimental Procedures.

### Chemicals

Upper axial cyano forms of cobalamin/pseudocobalamin were used for all B_12_-amendment experiments. Cyanocobalamin was purchased from Sigma-Aldrich UK. Cyanopseudocobalamin was prepared by guided biosynthesis from a culture of *Propionibacterium acidi-propionici* DSM 20273 as described previously and confirmed by UV-Vis, circular dichroism (CD), mass, and nuclear magnetic resonance (NMR) spectroscopic analysis [12].

### Strains and Growth Conditions

Details of microbial strains and culture conditions are provided in the Supplemental Experimental Procedures and Table S2.

### Molecular Methods

#### RNA Extraction and qRT-PCR

Total RNA was extracted [6] and treated with the Ambion Turbo DNase-Free Kit to remove genomic DNA. RNA was reverse transcribed into cDNA with SuperScript II (Invitrogen). Details of qRT-PCR are given in the Supplemental Experimental Procedures and Table S3.

#### Western Blotting

Total protein was extracted and western blot experiments performed as described in [32].

## Author Contributions

K.E.H., A.D.L., A.H., U.J.K., S.S., D.J.S., M.J.W., and A.G.S. designed the research; K.E.H., A.D.L., A.H., U.J.K., and S.S. performed the experiments; K.E.H., A.D.L., A.H., U.J.K., M.J.W., and A.G.S. analyzed the data; and K.E.H., D.J.S., B.K., M.J.W., and A.G.S. wrote the paper.

## Accession Numbers

The data described in this manuscript are available at https://www.repository.cam.ac.uk/handle/1810/254413.

## Supplemental Information

Document S1. Supplemental Experimental Procedures, Figures S1–S5, and Tables S1–S3

Data S1. Analysis of the Cobalamin Synthesis Pathway in Cyanobacteria and Identification of *bluB* in Rhizobiales and Rhodobacteria

Data S2. Identification of *COBT* and *COBS* in the Marine Microbial Eukaryote Transcriptome Sequencing Project

Document S2. Article plus Supplemental Information
