# Supplementary material for: Cyanobacteria and Eukaryotic Algae Use Different Chemical Variants of Vitamin B12
Source: Curr Biol. 2016 Apr 25;26(8):999–1008. doi: 10.1016/j.cub.2016.02.041 (PMC4850488; doi:10.1016/j.cub.2016.02.041)
Supplement: Document S1. Supplemental Experimental Procedures, Figures S1–S5, and Tables S1–S3 [file mmc1.pdf]

Current Biology, Volume *26*

Supplemental Information

Cyanobacteria and Eukaryotic Algae Use Different Chemical Variants of Vitamin B_{12}

Katherine Emma Helliwell, Andrew David Lawrence, Andre Holzer, Ulrich Johan Kudahl, Severin Sasso, Bernhard Kräutler, David John Scanlan, Martin James Warren, and Alison Gail Smith

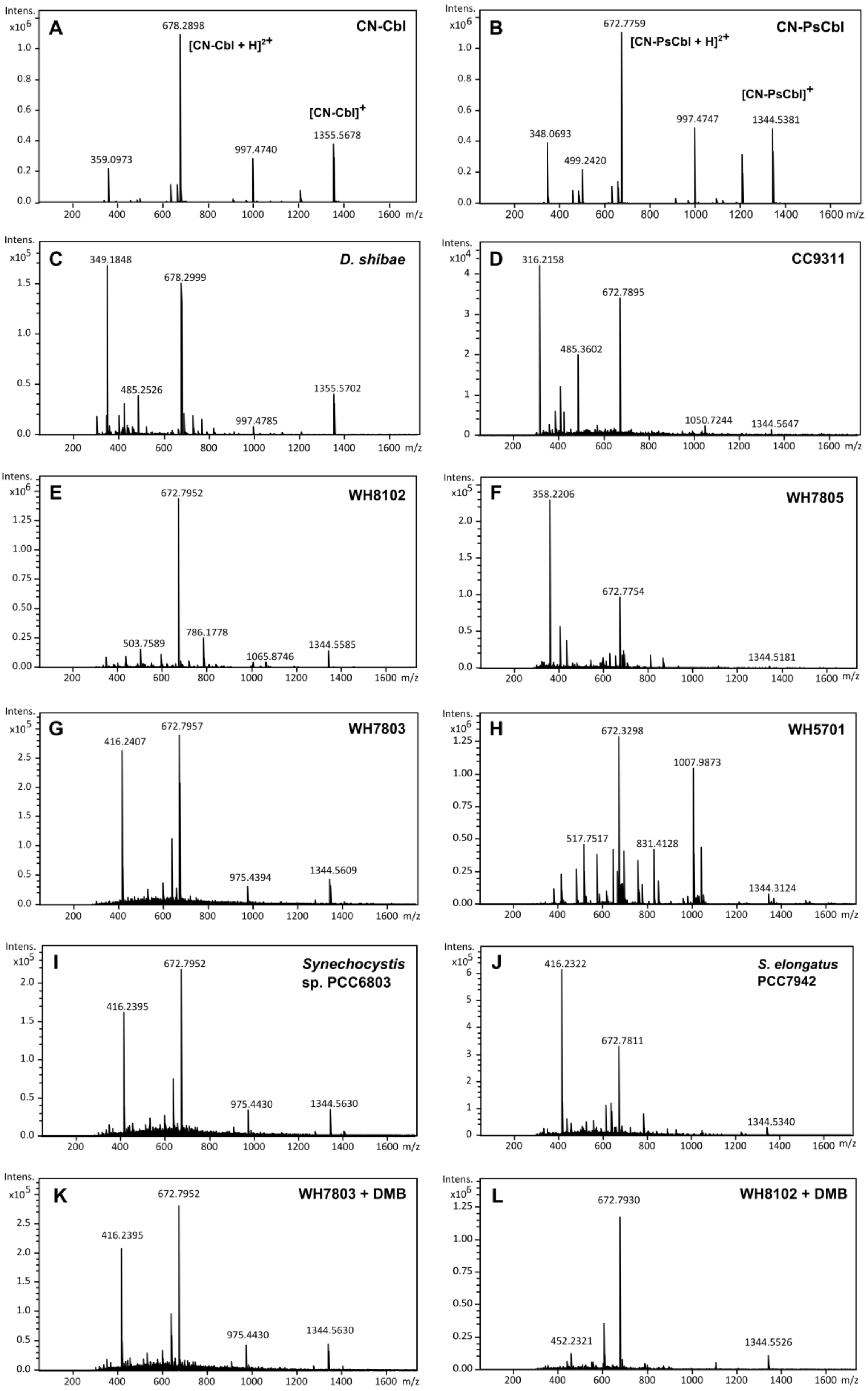

A
CN-Cbl
678.2898
[CN-Cbl + H]$^{2+}$
[CN-Cbl]$^{+}$
359.0973
997.4740
1355.5678
B
CN-PsCbl
672.7759
[CN-PsCbl + H]$^{2+}$
[CN-PsCbl]$^{+}$
348.0693
499.2420
997.4747
1344.5381
C
D. shibae
349.1848
678.2999
485.2526
997.4785
1355.5702
D
CC9311
316.2158
672.7895
485.3602
1050.7244
1344.5647
E
WH8102
672.7952
503.7589
786.1778
1065.8746
1344.5585
F
WH7805
358.2206
672.7754
1344.5181
G
WH7803
416.2407
672.7957
975.4394
1344.5609
H
WH5701
672.3298
1007.9873
517.7517
831.4128
1344.3124
I
Synechocystis sp. PCC6803
672.7952
416.2395
975.4430
1344.5630
J
S. elongatus PCC7942
416.2322
672.7811
1344.5340
K
WH7803 + DMB
672.7952
416.2395
975.4430
1344.5630
L
WH8102 + DMB
672.7930
452.2321
1344.5526
Intens.
m/z

Figure S1 ESI$^+$ mass spectra of B_{12} standards and B_{12} extracted from different bacteria. Related to Figure 1. **A.** cobalamin**;** **B.** pseudocobalamin; **C.** Alpha-proteobacterium *D. shibae*; **D.** *Synechococcus* sp. CC9311; **E.** *Synechococcus* sp. WH8102; **F.** *Synechococcus* sp. WH7805; **G.** *Synechococcus* sp. WH7803; **H**. *Synechococcus* sp. WH5701**;** **I.** *Synechocystis* sp. PCC6803*;* **J.** *Synechococcus elongatus* PCC7942; **K.** and **L.** *Synechococcus* sp. WH7803 and WH8103 respectively, grown with 1 µM DMB. Data from all cyanobacterial samples contain peaks which are characteristic of CN-pseudocobalamin with m/z 1344.5 $[CN\text{-}PsCbl]^+$ and m/z 672.7 $[CN\text{-}PsCbl + H]^{2+}$ but not m/z 1355.5 $[CN\text{-}Cbl]^+$ and m/z 678.3 $[CN\text{-}Cbl + H]^{2+}$ for CN-cobalamin. In contrast, *D. shibae* has the characteristic cobalamin ions.

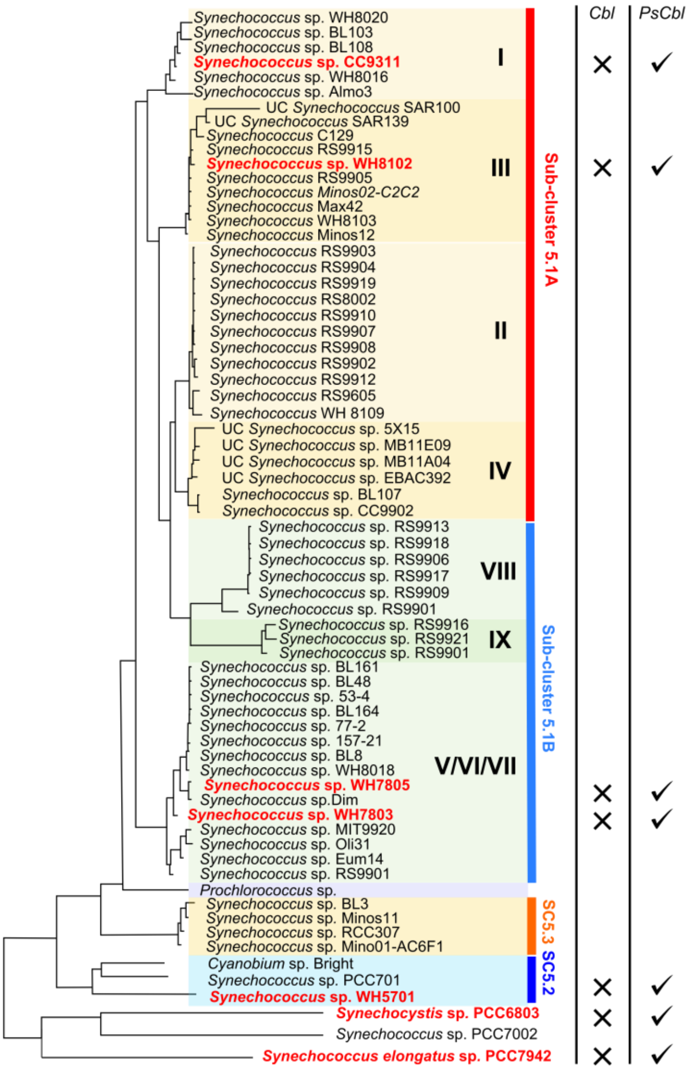

Figure S2 *Synechococcus* strains synthesise pseudocobalamin rather than cobalamin. Related to Figure 1. *Synechococcus* phylogeny adapted from [S1] indicating in red the strains surveyed in this study. Key: Cbl = synthesises cobalamin; PsCbl = synthesised pseudocobalamin.

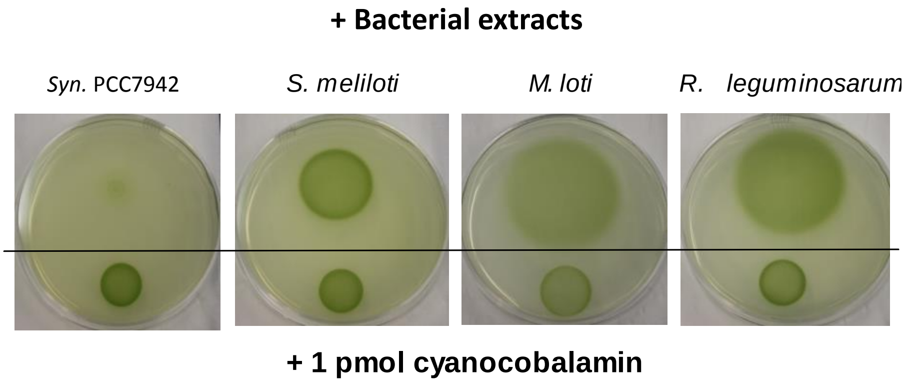

Figure S3 Growth of *L. rostrata* is supported by extracts of rhizobia but not cyanobacteria. Related to Figure 2. A lawn of *L. rostrata* cells was plated in top agar onto TAP solid medium, then 10 µl of different B_{12}-containing solutions i.e. freeze-dried extracts from different bacteria, each containing approximately 10 pmol of B_{12} (quantified using the *S. typhimurium* bioassay [S2]), were spotted onto the surface. The plates were left in the light for 10 days and then photographed. The cell extract from *Synechococcus elongatus* PCC7942 does not support growth, whereas a distinct lawn of algal cells is visible for each of the three rhizobial species *Sinorhizobium meliloti* 1021, *Mesorhizoium loti* MAFF303099 and *Rhizobium leguminosarum* 3841. The lower spot on each plate is from the addition of 1 pmol cobalamin standard.

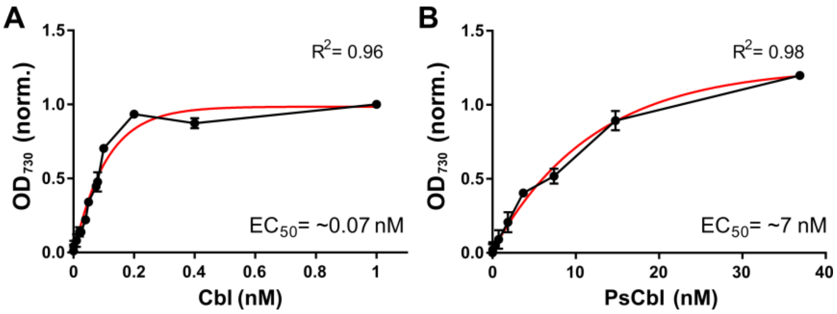

Figure S4 Dose response of *C. reinhardtii* B_{12}-dependent *metE* mutant [S3] with different B_{12} variants. Related to Figure 2. **A.** Cells were grown in TAP + Cbl for 96 hours and growth measured as OD_{730} incubated. **B**. As for (A) but cells were supplemented with PsCbl. OD_{730} was normalized to OD_{730} on 0.7 nM Cbl. Nonlinear fits were applied to the data and nutrient concentrations supporting half maximal biomass accumulation were estimated (EC_{50}) (mean ± SEM, *n*=3).

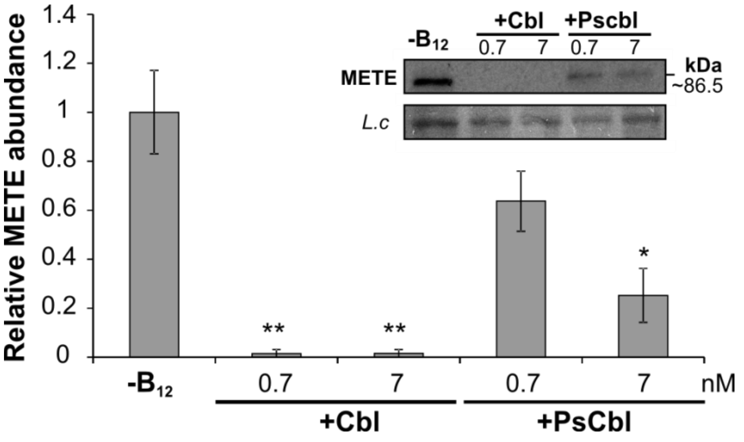

Figure S5. Western blot analysis of METE protein in *C. reinhardtii.* Related to Figure 3. Cells were grown with no supplementation or with 0.7 or 7 nM of Cbl or PsCbl, and total soluble protein extracted after 48 hours. Four µg of total protein was loaded for each sample. After transfer of protein to PVDF membrane and staining with Ponceau S, the blots were probed with a METE antibody [S4]. Band intensity analysis (integrated density) using ImageJ software [S5] allowed METE abundance to be quantified and normalised to the quantity of loaded protein from the stained blot (L.c: loading control). Mean ± SEM, n=3; *P ≤ 0.05, **P ≤ 0.01, ***P≤0.001 compared to the treatment without B_{12} (two-tailed Student's t test).

Table S1. Analysis of 13 sequenced algal genomes (*and one transcriptome) for proteins known in bacteria to be involved in DMB activation/lower ligand assembly. Related to Figures 4 and 5.

Searches of algal genomes utilised the Joint Genome Institute portal (http://genome.jgi.doe.gov/) or independent sources (*Ectocarpus siliculosus:* http://bioinformatics.psb.ugent.be/genomes/view/Ectocarpus-siliculosus, and *Cyanidioschyzon merolae*, http://merolae.biol.s.u-tokyo.ac.jp/). The data shown from *Pavlova lutheri* (strain name: RCC 1537, or alternatively CCAP 931/1) is from the Marine Microbial Eukaryote Transcriptome Sequencing Project [S6]. E-value and protein ID of retrieved sequences are given. All hits shown were verified via Pfam analysis to contain the expected conserved domains analogous to the relevant query protein used to carry out the BLASTP search (Supplemental Experimental Procedures), namely: nicotinate-nucleotide-DMB-phosphoribosyltransferase domain (CobT), Cobalamin-5-phosphate synthase domain (CobS) and Histidine phosphatase superfamily (branch 1) (CobC). Species shaded grey have been shown experimentally to require B_{12}.

**Group**	**Species (and genome version)**	Lower ligand assembly (**Early insertion pathway)**			Remodelling (*R.sphaeroides*)	
		CobT	*CobS*	*CobC*	*CbiZ*	*CbiB*
Rhodophyta	*Cyanidoschyzon merolae*	✘	✘	✓(5e-7; id: CMN085C)	✘	✘
Chlorophyta	*Chlamydomonas reinhardtii* (*v5.5*)	✓(6.7e-10; id: Cre12.g555500.t1.2)	✓(1.4e-5; id: Cre01.g036950.t1.2)	✓(2e-10; id: Cre10.g460300.t1.2)<br>✓(5.9e-9;id: Cre05.g232550.t1.2)<br>✓(2.5e-7; id: Cre13.g564000.t1.2)	✘	✘
	Volvox carteri f. *nagariensis* EVE (*v2*)	✘	✘	✓(6.8e-14; id:Vocar20015114m)	✘	✘
	Micromonas pusilla RCC299 (*v3*)	✘	✘	✓(4.4e-10; id:50990)	✘	✘
	Ostreococcus tauri OTH95(*v2*)	✘	✘	✘	✘	✘
	Coccomyxa sp. C-169 (*v2*)	✘	✘	✓(1.4e-12; id:14103)<br>✓(3.1e-9; id:28443)	✘	✘
	Chlorella variabilis NC64A	✘	✘	✘	✘	✘
Chromalveolata	*Pavlova lutheri* (strain name: RCC 1537, or alternatively CCAP 931/1)	✓<br>(1.4 e-17; 14947)	✓(9.9 e-15;2612)	✓(1.2e-6;1018)	✘	✘
	Emiliania huxleyi (CCMP1516) (*v1*)	✓(1.17e-26; id: 241304)	✓(3e-14; id: 95466)	✓(2.44e-8; id:213567)<br>✓(1.67e-8; id:223722)	✘	✘
	Phaeodactylum tricornutum CCAP1055/1 (*v2*)	✓(2.58e-25; id:16445)	✘	✘	✘	✘
	Fragilariopsis cylindrus CCMP 1102 (v1)	✓(2.22e-31; id:180280)	✘	✘	✘	✘
	Thalassiosira pseudonana (v3)	✘	✘	✓(7.27e-9; id:264296)	✘	✘
	Aureococcus anophagefferens clone 1984 (*v1*)	✓(3.12e-32; id:58669)	✘	✓(6.14e-0; id:71905)<br>✓(1.85e-6; id:14569)	✘	✘
	Ectocarpus siliculosus	✘	✘	✓(3e-11; id: Esi0119_0093)	✘	✘

Table S2: Strains, origin, and growth conditions for each alga grown during this study. Related to Figures 2, 4, 5 and 6.

Species	Media	Conditions	Origin
Algae			
Amphidinium carterae (CCAP 1102/6)	F/2[1]	25°C, standing, continuous light (20 µmol/m^2/sec)	A gift from Professor Christopher Howe, University of Cambridge, UK
Aureococcus anophagefferens (CCMP 1984)	L1[2]	18°C, standing, 16 h:8 h light:dark (20 µmol/m^2/sec)	Roscoff Culture Collection, France
Chlamydomonas reinhardtii (WT st.12) a wild-type strain derived from strain 137c)	TAP3	25°C, shaking 100 r.p.m, continuous light (120 µmol/m^2/sec)	A gift from Dr Saul Purton, UCL, London, UK
Chlamydomonas reinhardtii evolved B_{12}-dependent mutant (*metE*$^-$) (20) (in WT st.12 background)	TAP	25°C, shaking 100 r.p.m, continuous light (120 µmol/m^2/sec)	[S3]
Euglena gracilis	TAP	25°C, shaking, continuous light (100 µmol/m^2/sec)	A gift from Prof Alan Battersby, University of Cambridge, UK
Lobomonas rostrata (SAG 45/2)	TAP	25°C, shaking 100 r.p.m, continuous light (120 nmol/m^2/sec)	Experimental Phycology and Culture Collection of Algae at the University of Goettingen (EPSAG), Germany
Pavlova lutheri (strain name: RCC 1537, or alternatively CCAP 931/1)	F/2	25°C, standing, continuous light (20 µmol/m^2/sec)	A gift from Dr Craig Pooley, Swansea University, UK.
Ostreococcus tauri (OTH95)	L1	18°C, standing, 16 h:8 h light:dark (20 µmol/m^2/sec)	A gift from Dr Herve Moreau Oceanological Observatory of Banyuls-ser-Mer, France
Thalassiosira pseudonana (CCMP 1335)	F/2 + Si	18°C, standing, 16 h:8 h light:dark (20 µmol/m^2/sec)	A gift from Dr Richard Dorrell, Ecole Normale Supérieure, Paris, France
Phaeodactylum tricornutum (CCAP 1052/B)	F/2 + Si	25°C, shaking 120 r.p.m, continuous light (20 µmol/m^2/sec)	CCAP, Oban, UK
Cyanobacteria			
Synechococcus sp. WH8102, WH7803, WH7805, WH5701	ASW[4]	24°C, standing, continuous light (5 µmol/m^2/sec)	University of Warwick, UK
Synechococcus sp. CC9311	ASW +	24°C, standing, continuous light (5	University of Warwick, UK

	L1 trace metals	µmol/m^2/sec)	
Synechocystis sp. PCC 6803	BG11[5]	25°C, shaking 100 r.p.m, continuous light (100 µmol/m^2/sec)	CCAP, Oban, UK
Synechococcus elongatus PCC7942	BG11	25°C, shaking 100 r.p.m, continuous light (100 µmol/m^2/sec)	A gift from Dr David Lea-Smith, University of Cambridge, UK.

[1]f/2 [S7, S8]
[2]L1 [S9]

[3]TAP = Tris-Acetate-Phosphate medium (i.e. heterotrophic) [S10]
[4] ASW [S11]

[5] BG-11 [S12]

Table S3. Primers for qPCR analysis used in this study. Related to Figure 3.

Gene	Primer name	Sequence (5'→3')
C. reinhardtii		
METE	C.r_METE_qPCR_F5	GTTCCCCACCACAACCATC
	C.r_METE_qPCR_R5	CGATGAGGCCCTCGTACTC
SAHH	C.r SAHH_F1-	ACAACGCCGAGTTCAAGATT
	C.r SAHH_R1-	CCCATCACCTTCTTGGACAT
ACTIN	C.r ACTIN Exon2_F	CTGGCTTTGCTGGTGATGAT
	C.r ACTIN exon 3 R	CCAGAGTCCAGCACGATACC
UBQ	C.r UBQ2 3UTR F	GCGATTTCTCGTTGGGCAGT
	C.r UBQ2 3UTR R	TGGCCCATCCACTTGTCCTT
RACK1	C.r RCK1 exon 3 F	GCCACACCGAGTGGGTGTCGTGCG
	C.r RCK1 exon 4 R	CCTTGCCGCCCGAGGCGCACAGCG
P. tricornutum		
METE	P.tri METEb F-	TTTTGGATTGAGTCGCTGGGAA
	P.tri METEb R-	TGGTAGTTGCTCGTGATCCATT
CBA1	P.tri_aCBA1_F1	AGTCTTCGATTACCAGGCATCC
	P.tri_aCBA1_R1	TACCAACAACGGCACAAAAGTC
H4	P.tri HISTONE H4-F1	AGGTCCTTCGCGACAATATC
	P.tri HISTONE H4-R1	ACGGAATCACGAATGACGTT
RPS	P.tri RPS-F1	CGAAGTCAACCAGGAAACCAA
	P.tri RPS-R1	GTGCAAGAGACCGGACATACC
TBP	P.tri TBP-F1	ACCGGAGTCAAGAGCACACAC
	P.tri TBP-R1	CGGAATGCGCGTATACCAGT

Supplemental Experimental Procedures

Sequence similarity searches and analysis of putative orthologs

There are two pathways for B_{12} biosynthesis: the 'early cobalt-insertion' (also known as the anaerobic pathway) and 'late cobalt-insertion' (aerobic) pathways. Orthologues of genes encoding $\mathbf{B_{12}}$-synthesis enzymes, including BluB and the *bzaABCDE* operon, were searched for in sequenced cyanobacterial genomes using BLASTP [S13] and a set of template sequences (Dataset S1) as input sequences. The template sequences are derived from UniProt [S14] and have all been functionally verified. We used an e-value cut-off of $<10^{-5}$ and identified the most similar protein sequence for each of the template genes for the investigated species. We then used the rps-BLAST algorithm to identify protein domains for the best matching sequences from the TIGRFAM [S15], Conserved Domain Database [S16], Pfam [S17] and Clusters of Orthologous Groups of proteins [S18] databases. We compared the identified domains with a list of expected domains for each gene (Dataset S1), and used a threshold of at least one shared domain between the expected and identified domains as a requirement for the gene to be classified as a putative B_{12}-synthesis gene. In order to classify each species as either capable of *de novo* B_{12} synthesis or not, we used a threshold requiring that ≥ 15 genes from the early cobalt-insertion pathway to be present in a species for it to be classified as a B_{12} producer. At the time of the analysis (27th July, 2015) 123 sequenced cyanobacterial genomes were available in NCBI, representing the orders: Chroococcales, Gloeobacterales, Nostocales, Oscillatoriales, Pleurocapsales, Prochlorales and Stigonematales (Dataset S1).

Searches of algal genomes for orthologues of bacterial *cobT*, *cobS*, *cobC* (Table S1) utilised the Joint Genome Institute portal (http://genome.jgi.doe.gov/) or independent sources (*Ectocarpus siliculosus:* http://bioinformatics.psb.ugent.be/genomes/view/Ectocarpus-siliculosus, and *Cyanidioschyzon merolae,* http://merolae.biol.s.u-tokyo.ac.jp/). The origin and sequence IDs of the proteins that were used to perform BLASTP searches are as follows: CobT (*S. typhimurium*, gi: 1253537), CobS (*S. typhimurium*, gi: 20141415), CobC (*S. typhimurium*, gi: 20141373), CbiZ (*Rhodobacter sphaeroides*, gi: 488807154), CbiB (*S. typhimurium,* gi: 543942). To verify the identity of putative orthologous algal proteins we employed: 1) multiple sequence alignment using Jalview [S19] to check visually each gene model, 2) Pfam (27.0) analysis [S20] to identify conserved functional domains in each protein, including: Nicotinate-nucleotide-DMB-phosphoribosyltransferase domain (CobT), Cobalamin-5-phosphate synthase domain (CobS) and Histidine phosphatase superfamily (branch 1) (CobC), analogous to the relevant query protein used for BLASTP searches. For mining of the MMETSP dataset sequence similarity searches for CobT and CobS were carried out with *Emiliania huxleyi* proteins (protein id: 241304 and 95466, respectively). Subcellular localisation analysis was carried out on *C. reinhardtii* COBT and COBS using PredAlgo [S21] (according to the default settings) [S22].

Strains and growth conditions

The strains, origin, and growth conditions for each alga grown during this study are summarised in Table S2. Details of media used are from the Culture Collection of Algae and Protozoa, Oban, UK (http://www.ccap.ac.uk/). Growth assays were performed in 24-well plates using 2 mL medium supplemented with variable final concentrations of cyanocobalamin, cyanopseudocobalamin, and 5,6-dimethylbenzimidazole (DMB). Natural seawater was collected from coastal station L4 located in the western English Channel by Dr Glen Wheeler, Marine Biological Association (MBA), UK. Seawater was sterile-filtered directly after collection. Since bacteria are a potential source of B_{12}, cultures were either already axenic (*L. rostrata*), or treated with a cocktail of antibiotics to remove/reduce contaminating bacteria. *P. lutheri*, *T. pseudonana*, *A. carterae, E. gracilis* were grown for 6 days with ampicillin (1 mg/mL), kanamycin (1 mg/mL), and neomycin (0.25 mg/mL). The same cocktail of antibiotics was applied to *O. tauri* for 14-21 days with the addition of streptomycin (50 µg/mL). *C. reinhardtii* and *E. gracilis* were treated with kasaugamycin (75 µg/mL) and ampicillin (50 µg/mL). *A. anophagefferens* cells were exposed sequentially to penicillin (1 mg/mL), neomycin (0.25 mg/mL), streptomycin (0.10 mg/mL), and gentamicin (0.25 mg/mL) treatments, as previously described [S23]. For algal growth assays OD_{730} was measured to quantify growth. Regressions of optical density vs. microscopic cell counts were highly linear (R^2 > 0.97) for species investigated in this study. To monitor bacterial contamination cultures were regularly plated onto LB and TY plates (for freshwater algae) or ASW plates (+0.1% (w/v) glucose, 0.1% (w/v) yeast extract) for marine algae and marine *Synechococcus* strains, or YP plates for *Synechococcus elongatus* PCC7942 and *Synechocystis* sp. PCC6803. For qPCR analysis RNA samples were extracted from cultures of *C. reinhardtii* or *P. tricornutum* grown in TAP or F/2 media respectively in continuous white fluorescent light to mid/late log phase (3-7 days), with and without additions of cobalamin or pseudocobalamin.

Chemicals

Cyanopseudocobalamin was prepared by guided biosynthesis from a culture of *Propionibacterium acidi-propionici* DSM 20273 as described previously S[24], and confirmed by UV-Vis, CD, mass, and NMR spectroscopic analysis [S24].

LC-MS analysis of B_{12} variants

For B_{12} analysis by LC-MS, samples were separated on an Agilent 1100 series HPLC coupled to a Bruker micrOTOF-Q II mass spectrometer using an Ace 5 AQ column (2.1 x 150 mm; Advanced Chromatography Technologies) maintained at 30ºC and with a flow rate of 0.2 mL/min. The mobile phase consisted of 0.1% (v/v) formic acid in water (solvent A) and methanol (solvent B). The initial conditions were set at 95% A, 5% B. The concentration of solvent B was increased with a linear gradient to 70% over 25 min and back to the initial conditions at 30 min. The mass spectrometer was operated in the positive electrospray mode.

Reverse transcription quantitative real-time PCR (RT-qPCR)

Details of the primers and specific amplification conditions used for qPCR are given in Table S3. For each gene, primers were designed for different exons (i.e. they spanned an intron) so that genomic DNA contamination could be assessed. qPCR was carried out using Qiagen SYBR Green MasterMix using 10 ng of cDNA and 0.1 mM of each primer per reaction. Unique single products were verified by melt-curve analysis. No amplification was detected in no-RT control samples, confirming the absence of genomic DNA. Cycle threshold (C_T) values of two technical replicates were calculated using Rotor-Gene Q Software version 2.02, and primer efficiency was calculated with LinRegPCR [S25]. Quantification of each gene of interest was calculated based on the Pfaffl model [S26, S27], using the reference genes: Histone H4, 30S Ribosomal protein S1 (*RPS1*) and TATA box-binding protein (*TBP*) (*P. tricornutum*) [S28], and Actin (*ACT*), Receptor of activated *protein* kinase C 1 (*RACK1*) and Ubiquitin (*UBQ*) for *C. reinhardtii* [S29]. Results were subsequently verified using the relative expression software tool (REST, 2009) [S27].

***C. reinhardtii* reporter lines**

Reporter lines containing the *BLE.GFP* gene driven by the B_{12}-repressible *METE* promoter (Figure 6) were generated in *C. reinhardtii* st.12 background (Table S2) [S30]. To assess the effect of cobalamin versus pseudocobalamin on the *METE* promoter, growth assays were used to test resistance to zeocin in the different treatments. For each transformed line of *C. reinhardtii*, three replicate 'pre-cultures' were set up in 24-well plates on non-selective TAP medium (without zeocin) for each no addition/+Cbl/+PsCbl treatment. After 72 hours, 20 µL of these pre-cultures were inoculated into 2 mL for each treatment, this time with 20 µg/mL zeocin in 24-well plates. The OD_{730} of the culture was determined after 13 days to establish growth of the culture. Where cells had grown this indicated normal expression of the *BLE* gene conferring zeocin resistance. Where cells did not grow, this indicated that the added B_{12} variant had repressed expression of the *BLE* gene.

Supplemental References

S1. Scanlan, D. J, Ostrowski, M., Mazard, S., Dufresne, A., Garczarek, L., Hess, W.R., Post, A.F., Hagemann, M., Paulsen, I. and Partensky, F. (2009). Ecological genomics of marine picocyanobacteria. Microbiol. Mol. Biol. *Rev. 73* , 249–99.

S2. Raux, E., Lanois, A., Levillayer, F., Warren, M.J., Brody, E., Rambach, A. and Thermes, C. (1996). *Salmonella typhimurium* cobalamin (vitamin B_{12}) biosynthetic genes: functional studies in *S. typhimurium* and *Escherichia coli*. J. Bacteriol. *178*, 753–67

S3. Helliwell, K. E., Collins, S., Kazamia, E. K., Purton S. P., Wheeler, G. W., and Smith, A. G. (2014). Fundamental shift in vitamin B_{12} eco-physiology of a model alga demonstrated by experimental evolution. ISME J. *9*, 1446–1455

S4. Schneider, M. J., Ulland, M., and Sloboda, R. D. (2008).A protein methylation pathway in *Chlamydomonas* flagella is active during flagellar resorption. Mol. Biol. Cell. *19*, 4319–27.

S5. Schneider, C. A., Rasband, W. S., and Eliceiri, K. W. (2012). NIH Image to ImageJ: 25 years of image analysis. Nat. Methods *9*, 671–5.

S6. Keeling, P.J., Burki, F., Wilcox, H.M., Allam, B., Allen, E.E., Amaral-Zettler, L.A., Armbrust, E.V., Archibald, J.M., Bharti, A.K., Bell C.J., *et al*. (2014). The marine microbial eukaryote transcriptome sequencing project (MMETSP): illuminating the functional diversity of eukaryotic life in the oceans through transcriptome sequencing. PLoS Biol. *12*, e1001889.

S7. Guillard, R. R. L, and Ryther, J. H. (1962). Studies of marine planktonic diatoms: i. *Cyclotella nana hustedt*, and *Detonula confervacea* (cleve) gran. Can. J. Microbiol. *8*, 229–239.

S8. Guillard, R. R. L (1975) *Culture of Phytoplankton for Feeding Marine Invertebrates.* (Plenum Press, New York, USA) pp 20-25.

S9. Gorman, D. S., and Levine, R. P. (1965). Cytochrome f and plastocyanin: their sequence in the photosynthetic electron transport chain of *Chlamydomonas reinhardi*. Proc. Natl. Acad. Sci. U. S. A. *54*, 1665–9

S10. Guillard, R. R. L., and Hargraves, P. E. (1993). *Stichochrysis immobilis* is a diatom, not a chrysophyte. Phycologia 32, 234–236.

S11. Wilson, W. H., Carr, N. G., and Mann, N. H. (1996). The effect of phosphate status on the kinetics of cyanophage infection in the oceanic cyanobacterium *Synechococcus* sp. WH78031. J. Phycol. 32, 506–516.

S12. Rippka, R., Deruelles, J., Waterbury, J. B., Herdman, M., and Stanier, R. Y. (1979). Generic assignments, strain histories and properties of pure cultures of cyanobacteria. J. Gen. Microbiol. *111*, 1–61.

S13. Altschul, S.F., Madden, T.L., Schäffer, A.A., Zhang, J., Zhang, Z., Miller, W., Lipman, D.J. (1997). Gapped BLAST and PSI-BLAST: a new generation of protein database search programs. Nucleic Acids Res. *25*, 3389–402.

S14. The Uniprot Consortium UniProt: a hub for protein information. (2015). Nucleic Acids Res. *43*, D204–12

S15. Haft D.H., Selengut, J.D., Richter, R.A., Harkins, D., Basu, M.K., and Beck, E. (2013) TIGRFAMs and genome properties in 2013. Nucleic Acids Res. *41*, 387–395

S16. Marchler-Bauer, A., Derbyshire, M.K., Gonzales, N.R., Lu, S., Chitsaz, F., Geer, L.Y., Geer, R.C., He, J., Gwadz, M., Hurwitz, D.I., *et al.* (2014). CDD: NCBI's conserved domain database. Nucleic Acids Res. *43*, D222–D226.

S17. Finn, R.D., Bateman, A., Clements, J., Coggill, P., Eberhardt, R.Y., Eddy, S.R., Heger, A., Hetherington, K., Holm, L., Mistry, J., *et al.* (2014). Pfam: The protein families database. Nucleic Acids Res. *42*, 222–230.

S18. Galperin, M. Y., Makarova, K. S., Wolf, Y. I., and Koonin, E. V. (2014).Expanded microbial genome coverage and improved protein family annotation in the COG database. Nucleic Acids Res. *43*, D261–9.

S19. Waterhouse, A. M., Procter, J. B., Martin, D. M. A., Clamp, M., and Barton, G. J. (2009). Jalview Version 2--a multiple sequence alignment editor and analysis workbench. Bioinformatics *25*, 1189–91

S20. Finn, R.D., Tate, J., Mistry, J., Coggill, P.C., Sammut, S.J., Hotz, H.R., Ceric, G., Forslund, K., Eddy, S.R., Sonnhammer, E.L., *et al.* (2008). The Pfam protein families database. Nucleic Acids Res. *36*, D281–8.

S21. Tardif, M., Atteia, A., Specht, M., Cogne, G., Rolland, N., Brugière, S., Hippler, M., Ferro, M., Bruley, C., Peltier, G., *et al.* (2012). PredAlgo: a new subcellular localization prediction tool dedicated to green algae. Mol. Biol. Evol. *29*, 3625–39.

S22. Emanuelsson, O., Brunak, S., von Heijne, G., and Nielsen, H. (2007). Locating proteins in the cell using TargetP, SignalP and related tools. Nat. Protoc. *2*, 953–71.

S23. Berg, G. M., Repeta, D. J., and Laroche, J. (2002). Dissolved organic nitrogen hydrolysis rates in axenic cultures of *Aureococcus anophagefferens* (Pelagophyceae): comparison with heterotrophic bacteria. Appl. Environ. Microbiol. *68*, 401–404.

S24. Hoffmann, B., Oberhuber, M., Stupperich, E., Bothe, H., Buckel, W., Konrat, R., and Kräutler, B. (2000). Native corrinoids from *Clostridium cochlearium* are adeninylcobamides: spectroscopic analysis and identification of pseudovitamin $B(_{12})$ and factor A. J. Bacteriol. *182*, 4773–82.

S25. Ramakers, C., Ruijter, J. M., Deprez, R. H. L., and Moorman, A. F. M. (2003). Assumption-free analysis of quantitative real-time polymerase chain reaction (PCR) data. Neurosci. Lett. *339*, 62–6.

S26. Vandesompele, J., De Preter, K., Pattyn, F., Poppe, B., Van Roy, N., De Paepe, A., and Speleman, F. (2002). Accurate normalization of real-time quantitative RT-PCR data by geometric averaging of multiple internal control genes. Genome Biol. *3*, RESEARCH0034.

S27. Pfaffl, M. W., Horgan, G. W., and Dempfle, L. (2002). Relative expression software tool (REST) for group-wise comparison and statistical analysis of relative expression results in real-time PCR. Nucleic Acids Res. *30*, e36.

S28. Siaut, M., Heijde, M., Mangogna, M., Montsant, A., Coesel, S., Allen, A., Manfredonia, A., Falciatore, A., and Bowler C. (2007). Molecular toolbox for studying diatom biology in *Phaeodactylum tricornutum*. Gene *406*, 23–35.

S29. Mus, F., Dubini, A., Seibert, M., Posewitz, M. C., and Grossman, A. R. (2007). Anaerobic acclimation in *Chlamydomonas reinhardtii*: anoxic gene expression, hydrogenase induction, and metabolic pathways. J. Biol. Chem. *282*, 25475–86.

S30. Helliwell, K. E., Scaife, M. A., Sasso, S., Araujo, A. P. U., Purton, S., and Smith, A. G (2014). Unraveling vitamin B_{12}-responsive gene regulation in algae. Plant Physiol. *165*, 388–97.
