# Supplementary material for: Cyanobacteria and Eukaryotic Algae Use Different Chemical Variants of Vitamin B12
Source: Curr Biol. 2016 Apr 25;26(8):999–1008. doi: 10.1016/j.cub.2016.02.041 (PMC4850488; doi:10.1016/j.cub.2016.02.041)
Supplement: Document S2. Article plus Supplemental Information [file mmc4.pdf]

Current Biology

Article

Cyanobacteria and Eukaryotic Algae Use Different Chemical Variants of Vitamin B_{12}

Graphical Abstract

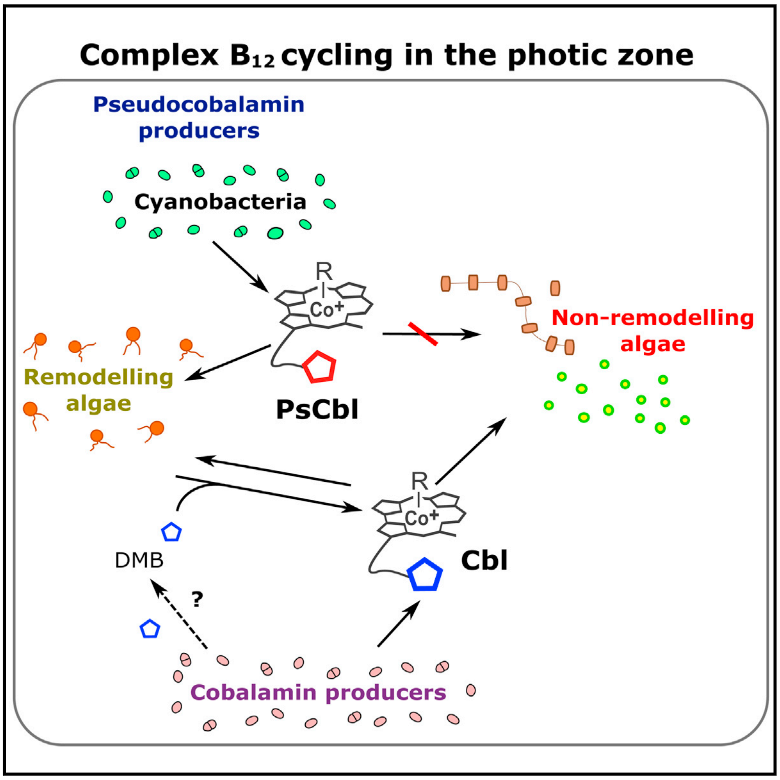

Highlights

- Dominant marine cyanobacteria synthesize only pseudocobalamin
- Pseudocobalamin is orders of magnitude less bioavailable to eukaryotic algae
- Certain algae can remodel pseudocobalamin to a bioavailable form
- This implies a complex B_{12} cycle between microbes in the photic zone

Authors

Katherine Emma Helliwell, Andrew David Lawrence, Andre Holzer, ..., David John Scanlan, Martin James Warren, Alison Gail Smith

Correspondence

katherine.helliwell@mba.ac.uk (K.E.H.), as25@cam.ac.uk (A.G.S.)

In Brief

Helliwell et al. demonstrate that the two major groups of photosynthetic microbes in the photic zone, cyanobacteria and microalgae, use different forms of vitamin B_{12}. These findings challenge the oversimplified assumption of a linear flux of B_{12} from producers to consumers, in favor of a more complex network of B_{12} production, uptake, and cycling.

Helliwell et al., 2016, Current Biology *26*, 999–1008
April 25, 2016 ©2016 The Authors
http://dx.doi.org/10.1016/j.cub.2016.02.041

CrossMark
CellPress

Current Biology
Article

CellPress

Cyanobacteria and Eukaryotic Algae Use Different Chemical Variants of Vitamin B_{12}

Katherine Emma Helliwell,[1,6,*] Andrew David Lawrence,[2] Andre Holzer,[1,3] Ulrich Johan Kudahl,[1] Severin Sasso,[1,7] Bernhard Kräutler,[4] David John Scanlan,[5] Martin James Warren,[2] and Alison Gail Smith[1,*]
[1]Department of Plant Sciences, University of Cambridge, Cambridge CB2 3EA, UK
[2]School of Biosciences, University of Kent, Kent CT2 7NJ, UK
[3]Institute of Pharmacy and Molecular Biotechnology, Ruprecht-Karls University Heidelberg, Im Neuenheimer Feld 364, 69120 Heidelberg, Germany
[4]Institute of Organic Chemistry and Centre of Molecular Biosciences, University of Innsbruck, Innrain 80/82, 6020 Innsbruck, Austria
[5]School of Life Sciences, University of Warwick, Coventry CV4 7AL, UK
[6]Present address: Marine Biological Association of the UK, Citadel Hill, Plymouth PL1 2PB, UK
[7]Present address: Institute of General Botany and Plant Physiology, Friedrich Schiller University, 07743 Jena, Germany
*Correspondence: katherine.helliwell@mba.ac.uk (K.E.H.), as25@cam.ac.uk (A.G.S.)
http://dx.doi.org/10.1016/j.cub.2016.02.041

SUMMARY

Eukaryotic microalgae and prokaryotic cyanobacteria are the major components of the phytoplankton. Determining factors that govern growth of these primary producers, and how they interact, is therefore essential to understanding aquatic ecosystem productivity. Over half of microalgal species representing marine and freshwater habitats require for growth the corrinoid cofactor B_{12}, which is synthesized de novo only by certain prokaryotes, including the majority of cyanobacteria. There are several chemical variants of B_{12}, which are not necessarily functionally interchangeable. Cobalamin, the form bioavailable to humans, has as its lower axial ligand 5,6-dimethylbenzimidazole (DMB). Here, we show that the abundant marine cyanobacterium *Synechococcus* synthesizes only pseudocobalamin, in which the lower axial ligand is adenine. Moreover, bioinformatic searches of over 100 sequenced cyanobacterial genomes for B_{12} biosynthesis genes, including those involved in nucleotide loop assembly, suggest this is the form synthesized by cyanobacteria more broadly. We further demonstrate that pseudocobalamin is several orders of magnitude less bioavailable than cobalamin to several B_{12}-dependent microalgae representing diverse lineages. This indicates that the two major phytoplankton groups use a different B_{12} currency. However, in an intriguing twist, some microalgal species can use pseudocobalamin if DMB is provided, suggesting that they are able to remodel the cofactor, whereas *Synechococcus* cannot. This species-specific attribute implicates algal remodelers as novel and keystone players of the B_{12} cycle, transforming our perception of the dynamics and complexity of the flux of this nutrient in aquatic ecosystems.

INTRODUCTION

Eukaryotic microalgae are photosynthetic microbes estimated to be responsible for up to 50% of global carbon fixation [1]. Elucidation of factors that control algal community structure and dynamics is thus fundamental to understanding the global cycling of carbon. Nutrients such as iron, nitrogen, and phosphorus clearly play an important role [2], but many microalgae also require the vitamins B_1 (thiamine), B_7 (biotin), or B_{12} for growth [3]. B_{12} is required as a cofactor for methionine synthase (METH; EC 2.1.1.13) activity, a key enzyme of cellular one-carbon (C1) metabolism important for production of the universal methyl donor S-adenosylmethionine (SAM), and for folate cycling necessary for DNA synthesis [4]. Those algae that do not need a supply of B_{12} cannot synthesize the vitamin; rather, they possess an alternative form of methionine synthase (METE; EC 2.1.1.14) that can catalyze the same reaction in a B_{12}-independent fashion [5, 6].

Measurement of B_{12} levels in the water column have indicated concentrations of ~10 pM in freshwater ecosystems [7] and are often below the threshold of detection in certain marine habitats, including large areas of the northeast Pacific margin [8]. The scarcity of this micronutrient is therefore thought to limit phytoplankton abundance [8], so competition for B_{12} among those organisms that require/use it is likely. Indeed, field-enrichment experiments found that, whereas N addition stimulated all microbial growth, there was a specific growth enhancement of phytoplankton >5 μm (i.e., the larger eukaryote fraction) with B_{12} supplementation [9]. However, several recent studies have demonstrated that heterotrophic bacteria can satisfy microalgal requirements for B_{12} via mutualistic interactions (e.g., [5, 10]).

Provision from prokaryotes is particularly pertinent because the biosynthetic pathway of this cofactor is confined to certain archaea and bacteria. B_{12} is an umbrella term that refers to cobalt-containing corrinoids (ring-contracted tetrapyrroles), which have upper and lower axial ligands to the cobalt ion (Figure 1A). The nature of these ligands varies, leading to diversity in the structural forms of B_{12}. Methylcobalamin, where the upper axial ligand is a methyl group, is involved in methyl-transfer

CrossMark

This is an open access article under the CC BY license (http://creativecommons.org/licenses/by/4.0/).

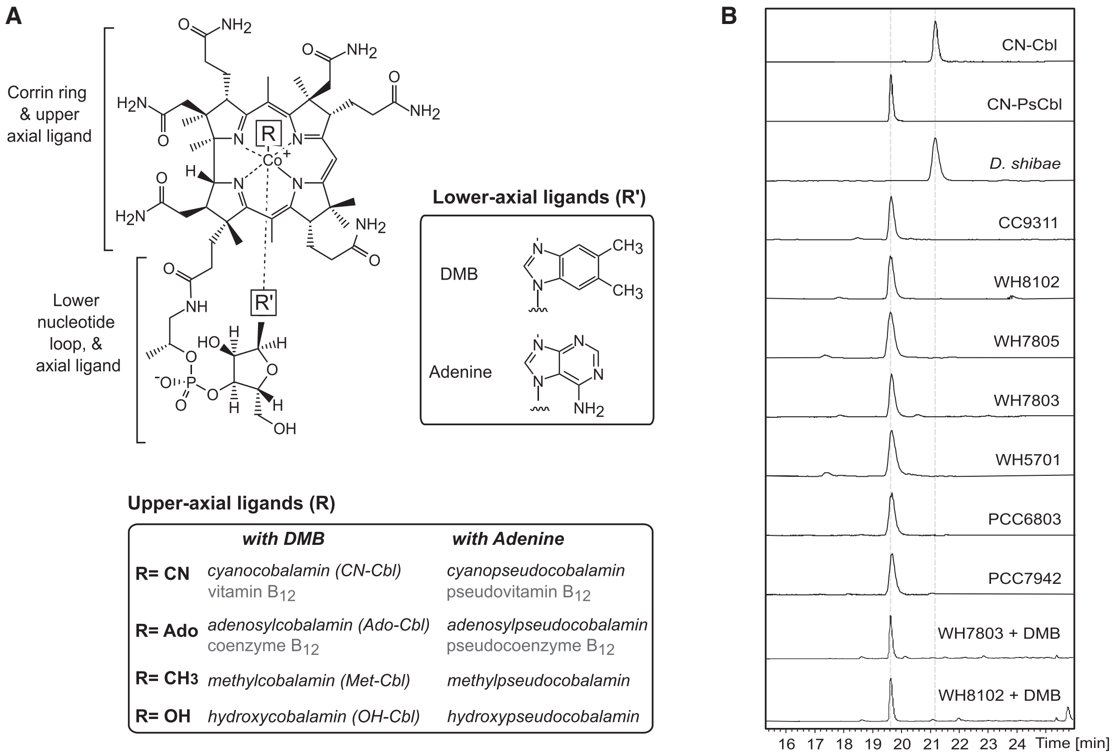

	with DMB	*with Adenine*
R= CN	*cyanocobalamin (CN-Cbl)* vitamin B_{12}	*cyanopseudocobalamin* pseudovitamin B_{12}
R= Ado	*adenosylcobalamin (Ado-Cbl)* coenzyme B_{12}	*adenosylpseudocobalamin* pseudocoenzyme B_{12}
R= CH_3	*methylcobalamin (Met-Cbl)*	*methylpseudocobalamin*
R= OH	*hydroxycobalamin (OH-Cbl)*	*hydroxypseudocobalamin*

Figure 1. *Synechococcus* Strains Synthesize Pseudocobalamin Rather Than Cobalamin

(A) Structural variants of B_{12}.

(B) HPLC-MS extracted ion chromatograms for m/z 1355.5 cyanocobalamin and m/z 1344.5 cyanopseudocobalamin (see also Figure S1). The lower tracks display the chromatograms for cell-free extracts derived from cultures of cyanobacterial species from Figure S2. Experiments were carried out in triplicate, and one replicate representative of each strain is shown.

reactions, whereas adenosylcobalamin (coenzyme B_{12}) is used for radical-based rearrangements and reductions [11]. The identity of the base found as the lower axial ligand, bound via a nucleotide loop, may vary too. In cobalamin, the base is 5, 6-dimethylbenzimidazole (DMB). Many bacteria, including methanogens and anaerobes such as *Clostridium* species, synthesize a B_{12} vitamer known as pseudocobalamin [12, 13], which differs from cobalamin in that DMB is replaced by adenine. Intrinsic factor, the mammalian B_{12}-binding protein important in uptake from the gut, has a lower affinity for pseudocobalamin than cobalamin [14]. Pseudocobalamin is therefore considered not "bioavailable" to humans, and the efficacy of vitamin supplements produced from cyanobacteria such as *Spirulina* and *Aphanizomenon*, which also contain pseudocobalamin, has been questioned [15, 16].

Cyanobacteria are the numerically dominant photosynthetic microbes in the marine environment [17]. Two cyanobacterial strains, *Crocosphaera watsonii* WH8501 and *Synechococcus* sp. WH7803, were reported to release B_{12} into the media at rates exceeding those estimated for the heterotrophic bacterium *Halomonas*, suggesting that cyanobacteria might be the major source of B_{12} for marine algae [18]. However, indications from the early literature suggest consideration of algal specificity toward different B_{12}-like factors may be pertinent [19]. Here, we investigate corrinoids in several strains of *Synechococcus*, an abundant and ubiquitous marine cyanobacterium [17, 20]; the nature of their axial ligands; and their ability to support growth of eukaryotic primary producers.

RESULTS

Synechococcus Species Make Only Pseudocobalamin

The biosynthesis of the corrinoid ring of B_{12} from the common tetrapyrrole progenitor uroporphyrinogen III requires at least 20 enzymatic steps, and several routes are known [21]. In a preliminary investigation, Sañudo-Wilhelmy et al. [22] searched for the presence of B_{12}-biosynthesis genes in ~40 marine cyanobacteria with sequenced genomes. They found that all but one species had putative homologs for at least 11 of these genes and so concluded that they were capable of making B_{12}. However, they did not investigate the genes involved in synthesis of the lower axial ligand and so could not conclude whether the cyanobacteria studied produced cobalamin or pseudocobalamin. To refine the analysis of B_{12}-biosynthesis genes, we searched 123 sequenced cyanobacterial genomes for all 20 genes of the corrinoid pathway. All but six species contained at least 15/20 of these genes and were predicted to be B_{12} producers (Data S1; Supplemental Experimental Procedures). Additionally, we searched for genes involved in DMB biosynthesis, for which two routes are currently known [23, 24]. The BluB enzyme, first characterized in *Sinorhizobium meliloti* (Rhizobia) makes DMB from riboflavin under aerobic

conditions [23]. Using this sequence as a query, no hits were found in 118 cyanobacterial genomes including *C. watsonii* WH8501 and *Synechococcus* sp. WH7803. For five species (including three from the *Fischerella* genus), hits for BluB were obtained: two were annotated as cob(II)yrinic acid *a,c*-diamide reductase (CobR), which is an enzyme of an earlier stage of B_{12} biosynthesis, whereas the others were unknown. In contrast, BluB homologs were found in 80% of sequenced rhizobia (227/284 genomes; Data S1C) and 60% of Rhodobacterales species (77/128; Data S1D) including *Mesorhizobium loti*, *Sinorhizobium meliloti*, *Rhizobium leguminosarum*, and the marine bacterium *Dinoroseobacter shibae*, all of which can support algal B_{12}-auxotrophic growth [10, 25]. More recently, a second route for DMB biosynthesis was identified in the obligate anaerobic bacterium *Eubacterium limosum* [24], and enzymes encoded by the *bzaABCDE* operon were shown to direct DMB production via an oxygen-sensitive reaction from the purine precursor 5-aminoimidazole ribotide (AIR) [24]. We found that none of the cyanobacterial genomes encoded the full *bzaABCDE* operon (Data S1B). Moreover, CobT, which is required for DMB activation, is absent from all but two cyanobacterial genomes. Taken together, these searches suggest that the vast majority of cyanobacteria cannot make DMB.

To validate the observations from the bioinformatics analysis, we wanted to assess directly what corrinoids are synthesized by cyanobacteria so investigated the B_{12} content of strains of marine *Synechococcus*, because this is an ancient and ecologically abundant lineage [20] with a mean global abundance of $7.0 \pm 0.3 \times 10^{26}$ cells y^{-1}, high-biomass-specific CO_2 fixation rates [17, 26] and axenic strains are available. Corrinoids can be extracted from cells as their cyano-derivatives and then analyzed by high-performance liquid chromatography (HPLC)-mass spectrometry (LC-MS). First, using purified cyanocobalamin (obtained commercially) and cyanopseudocobalamin, prepared from *Propionibacterium acidi-propionici* [12], we were able to distinguish the two variants of B_{12} by their different retention times on the LC (Figure 1B) and different mass (Figures S1A and S1B). Derivatized cell lysate obtained from axenic cultures of the heterotrophic marine bacterium *D. shibae* DFL12T contained only cyanocobalamin. We next tested five members of the *Synechococcus* lineage representing different clades and habitats (highlighted in red in Figure S2): coastal strain CC9311 (sub-cluster [SC] 5.1A; clade I); oligotroph WH8102 (SC5.1A; clade III); WH7803 and WH7805 (SC5.1B; clades V and VI, respectively) which are widely distributed in various oceanic waters; and the estuarine strain WH5701 (SC5.2). A single peak was observable in these samples at a retention time consistent with the pseudocobalamin standard (Figure 1B), and its identity was confirmed by MS (Figures S1D–S1H). To facilitate subsequent physiological work, we also tested two model freshwater cyanobacterial species *Synechocystis* sp. PCC6803 and *Synechococcus elongatus* PCC7942, because these species grow quickly and easily in the laboratory. Again, cell lysates from these strains contained only pseudocobalamin (Figures 1B, S1I, and S1J). Together, these data demonstrate that the cyanobacterial species sampled here make only pseudocobalamin in axenic laboratory culture conditions.

However, some B_{12}-synthesizing bacteria can modify endogenous B_{12} forms with an alternative base [27]. For instance, although *Salmonella enterica* cannot make DMB, it can import it and then make cobalamin instead of pseudocobalamin [27]. To investigate whether *Synechococcus* can perform this so-called "guided biosynthesis," we grew strains WH8102 and WH7803 (representing SC5.1A and 5.1B) in the presence of pseudocobalamin and 1 μM DMB, but only pseudocobalamin was detected (Figures 1B, S1K, and S1L). We conclude therefore that *Synechococcus* cannot replace the adenine base with DMB to make cobalamin.

Pseudocobalamin Is Orders of Magnitude Less Bioavailable to Eukaryotic Algae

We next tested whether cyanobacterially derived B_{12} could be utilized by eukaryotic algae. Cell-free extracts of *S. elongatus* PCC7942 (pseudocobalamin producer) were unable to rescue growth of the B_{12}-dependent freshwater alga *Lobomonas rostrata*, whereas there is clear growth with the addition of extracts of three rhizobial bacteria (Figure S3), which all encode BluB [28]. It is conceivable that the growth is due to other compounds in the crude lysate, so this initial experiment was extended using the purified compounds, cyanocobalamin and cyanopseudocobalamin. Equivalent concentrations of each B_{12} variant were added to axenic cultures of B_{12}-dependent microalgae from different algal lineages: marine species *Ostreococcus tauri* (Chlorophyta, Mamiellophyceae); *Amphidinium carterae* (Alveolata, Dinoflagellate); *Pavlova lutheri* (Haptophyta, Prymnesiophyceae); *Thalassiosira pseudonana* (Heterokontophyta, Coscinodiscophyceae); *Aureococcus anophagefferens* (Heterokontophyta, Pelagophyceae); and the freshwater species *Euglena gracilis* (Excavata, Euglenozoa) and *L. rostrata* (Chlorophyta, Volvocales). We also tested a B_{12}-dependent *metE* mutant of *Chlamydomonas reinhardtii* (Chlorophyta, Volvocales) [29]. When pseudocobalamin was supplied at a concentration of 0.07 or 0.7 nM, we observed little or no growth in any of the marine species, nor with the *C. reinhardtii metE* mutant or *L. rostrata*. This is in contrast to cobalamin, which supported growth of all algal cultures at equivalent concentrations (Figure 2; Student's t test; $p < 0.05$; $n = 3$). For *O. tauri*, *A. carterae*, and *T. pseudonana*, and to a lesser extent the *C. reinhardtii metE* mutant, provision of pseudocobalamin at 7 nM (~10 μg/l) supported growth to a similar extent as cobalamin (Figures 2A, 2B, 2D, and 2H), although this amount is significantly higher than found in natural ecosystems (with reported concentrations ranging from below the detection threshold to 0.03 nM across large areas of the northeast Pacific margin, for instance) [7, 8]. One way to compare the efficacy of the different B_{12} variants is to carry out dose-response experiments, which enable determination of an EC_{50} (that is the effective concentration required to support half-maximal biomass accumulation) and also provide an indication of the minimum amount needed to observe any growth, and so we carried these out with the *C. reinhardtii metE* mutant. Figure S4 shows that the EC_{50} was ~0.07 nM for cobalamin, compared to ~7 nM (~100-fold higher) for pseudocobalamin. In addition, it is clear that even the highest concentration of pseudocobalamin used (40 nM) is not saturating, whereas 0.2 nM cobalamin supports maximum growth. For *E. gracilis*, some growth was observed even at the lowest pseudocobalamin concentration, but it was still significantly lower than with cobalamin (Figure 2G). Thus, pseudocobalamin is orders of magnitude less

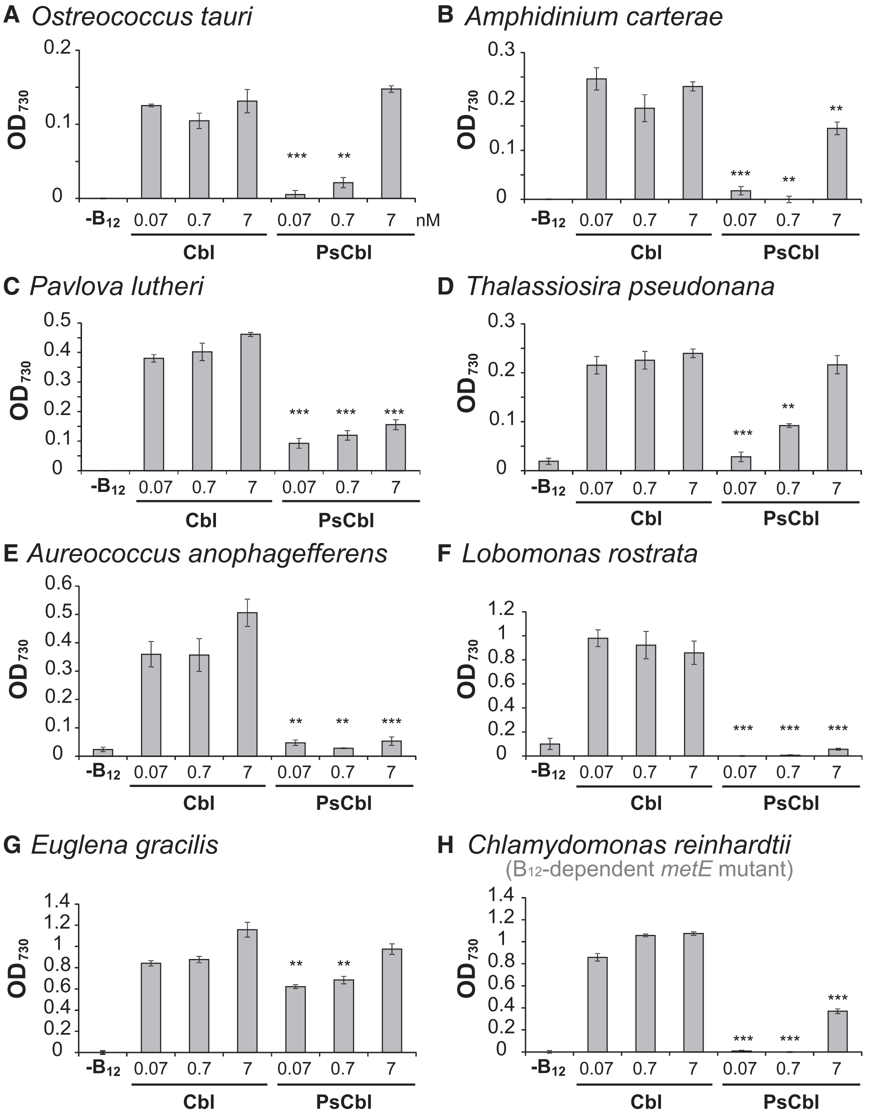

Figure 2. Pseudocobalamin Poorly Supports Growth of B_{12}-Dependent Eukaryotic Algae

(A–H) Growth yield (OD_{730}) of algae in liquid medium supplemented with or without cyanocobalamin or cyanopseudocobalamin (at 0.07, 0.7, or 7 nM) in batch culture after at least two transfers (until the cells had died in the $-B_{12}$ treatment). (A) *O. tauri*, (B) *A. carterae*, (C) *P. lutheri*, (D) *T. pseudonana*, (E) *A. anophagefferens*, (F) *L. rostrata*, (G) *E. gracilis*, and (H) *C. reinhardtii* B_{12}-dependent *metE* mutants are shown [29]. Cbl, cyanocobalamin; PsCbl, cyanopseudocobalamin. *$p \leq 0.05$; **$p \leq 0.01$; ***$p \leq 0.001$ compared with the equivalent concentration of Cbl (two-tailed Student's t test; mean ± SEM; n = 3). See also Figures S3 and S4.

bioavailable to eukaryotic algae. It is notable to mention that *E. gracilis* has also been demonstrated to encode a B_{12}-dependent (type II) ribonucleotide reductase [30], which could account for the growth response to pseudocobalamin observed in this alga.

We reasoned that the reduced ability of pseudocobalamin to support growth of algal B_{12}-auxotrophs may be either because the molecule cannot be used as a cofactor or because it does not get transported into algal cells. To investigate the latter possibility, we took advantage of the presence of B_{12}-responsive genes previously identified in the marine diatom *Phaeodactylum tricornutum* and *C. reinhardtii* [31, 32]. These algae do not need B_{12} for growth but will uptake and use it if it is available [5, 6]. Several genes in these algae are responsive to B_{12}: *METE* (in *P. tricornutum* and *C. reinhardtii*) [6, 31, 32]; *CBA1*, encoding a novel cobalamin acquisition protein (in *P. tricornutum* only) [31]; and S-adenosylhomocysteine hydrolase, *SAHH* (in *C. reinhardtii* only) [32]. Using qRT-PCR, we analyzed their expression in cells grown in the presence of 0.7 nM cobalamin or pseudocobalamin. For *P. tricornutum*, both forms of B_{12} resulted in downregulation of *METE,* but the effect was less pronounced with pseudocobalamin compared to that with cobalamin (Student's t test; $p < 0.001$; n = 3; Figure 3A). As previously demonstrated, cobalamin suppressed *CBA1* [31], but this gene was significantly upregulated by pseudocobalamin (Student's t test; $p < 0.05$; n = 3; Figure 3A). In *C. reinhardtii*, both *METE* (Student's t test; $p < 0.05$; n = 3) and *SAHH* (Student's t test; $p < 0.01$; n = 3) were downregulated relative to the no supplementation control (Figure 3B) with both forms of B_{12}. Subsequent western blot analysis using polyclonal antibodies against *C. reinhardtii* METE protein [32] demonstrated a modest reduction of METE abundance when cells were grown with pseudocobalamin, although not to the same extent as cobalamin (Figure S5). Nonetheless, the effect of pseudocobalamin on the expression of these four B_{12}-responsive genes indicates that the molecule can enter both *C. reinhardtii* and *P. tricornutum* cells.

Certain Algae Are Capable of Remodeling Pseudocobalamin

By analogy with the guided biosynthesis described earlier, some bacteria that cannot synthesize B_{12} de novo can modify imported forms with an alternative base, via "corrinoid remodeling" [33, 34]. To investigate this possibility in algae, we grew B_{12}-requiring species in the presence of pseudocobalamin and a range of DMB concentrations. For most, growth was not restored by DMB supplementation (Figures 4A, 4B, and 4D–4G). However, for *P. lutheri* and the *C. reinhardtii metE* mutant, addition of DMB alongside pseudocobalamin rescued growth to the same extent as cobalamin (Figures 4C and 4H). A dose-response experiment with *P. lutheri* established an EC_{50} value of ~18 pM for cobalamin (Figure 5A). A similar experiment with

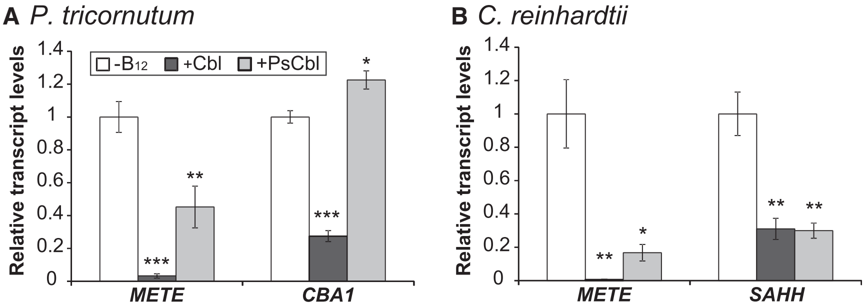

Figure 3. Pseudocobalamin Affects Expression of B_{12}-Responsive Genes in *P. tricornutum* and *C. reinhardtii*

(A and B) qRT-PCR analysis of (A) *METE* and *CBA1* expression in *P. tricornutum* and (B) *METE* and *SAHH* expression in *C. reinhardtii,* without B_{12} or with 0.7 nM Cbl/PsCbl. Expression was normalized using three housekeeping genes: *Histone H4*; 30S, ribosomal protein S1, *RPS*; and TATA box-binding protein, *TBP* for *P. tricornutum* and receptor of activated *protein* kinase C 1, *RACK 1*; Actin, *ACT*; ubiquitin, *UBQ* for *C. reinhardtii*. *p $\leq$ 0.05; **p $\leq$ 0.01; ***p $\leq$ 0.001 compared to the $-B_{12}$ treatment (two-tailed Student's t test; mean ± SEM; n = 3). See also Figure S5.

a fixed concentration (0.7 nM) of pseudocobalamin but varying the amount of DMB revealed a similar EC_{50} (~23 pM; Figure 5B). Interestingly, an equivalent dose-response curve (and EC_{50} value: ~26 pM) was observed when cells were grown in medium made using natural filtered seawater rather than artificial sea salts. Thus, the level of DMB in the natural filtered seawater is not sufficient to allow remodeling; otherwise, the dose-response curve would be shifted to the left. That comparable levels of B_{12} and DMB (at a fixed level of pseudocobalamin) are able to rescue B_{12}-dependent growth implies that *P. lutheri* is remodeling pseudocobalamin with DMB to generate cobalamin. Dosage experiments with the *C. reinhardtii* B_{12}-dependent *metE* mutant also identified similar EC_{50} values of ~28 pM and ~70 pM for cobalamin and DMB, respectively (Figures 5C and 5D). In this case, the EC_{50} value for DMB was slightly higher than cobalamin. We also tested whether *C. reinhardtii* is capable of de novo lower-loop synthesis and grew the *C. reinhardtii* B_{12}-dependent *metE* mutant with DMB, alongside (dicyano)cobinamide, a B_{12} precursor that lacks the DMB ribonucleotide tail, but no restoration of growth was observed (Figure 6A).

Our data indicate that, of eight diverse algal species studied, six do not appear to be able to use exogenous DMB. Nevertheless, the observation that growth of the *C. reinhardtii* B_{12}-dependent *metE* mutant (alongside that of *P. lutheri*) with pseudocobalamin is restored by DMB provision suggests that these algae are able to chemically modify pseudocobalamin to a form that can support B_{12}-auxotrophic growth. To test more directly whether pseudocobalamin is being remodeled, we grew samples of *C. reinhardtii* in the presence of (1) cobalamin, (2) pseudocobalamin (1 nM), and (3) pseudocobalamin (1 nM) + DMB (1 μM) and prepared cell lysate for LC-MS analysis. However, we could not detect any form of B_{12} from lysed cells. We infer from this that intracellular B_{12} levels are extremely low, i.e., the quantity from ~1×10^{10} cells is below the threshold detection of the LC-MS (which in our system is ~1×10^{12} molecules). Without a clear idea of what order of magnitude more biomass would be required and constrained by the limitations of scale, we turned to alternative means of characterizing remodeling activity and investigated the effect of DMB + pseudocobalamin on gene expression in *C. reinhardtii.* Previously, we had generated several transgenic lines of *C. reinhardtii* expressing the B_{12}-responsive element of the *METE* gene fused to the *BLE* gene, which confers resistance to the antibiotic Zeocin [32]. This reporter gene construct enables rapid and easy measurement of B_{12}-responsive gene expression, whereby growth with cobalamin represses expression of *BLE* so that cells die in the presence of Zeocin (Figure 6B). In contrast, pseudocobalamin alone had little effect, but the inclusion of DMB impaired growth to the same extent as cobalamin, demonstrating that *C. reinhardtii* converts DMB and pseudocobalamin into a form that is able to repress the *METE* promoter.

The pathway for pseudocobalamin remodeling has been investigated previously in the purple bacterium *Rhodobacter sphaeroides*, and cobinamide amidohydrolase (CbiZ) and cobinamide-phosphate synthase (CbiB) have been implicated in this process [34]. We could not identify *cbiZ* or *cbiB* in any of the algal genomes we analyzed. We therefore searched for proteins shown to be involved in lower-loop assembly and activation [34] in *S. enterica* (CobT, CobS, and CobC) [27], where mutants of CobT are unable to incorporate exogenous DMB (Figure 6C). We identified genes encoding all three of these proteins in *C. reinhardtii*, which exhibits the remodeling phenotype (Table S1; Figure 5). In contrast, BLASTP searches of the genomes of *O. tauri* and *T. pseudonana*, species that do not appear to remodel, were negative for CobT and CobS. Although a hit for CobC was identified in *T. pseudonana*, it should be noted that CobC catalyzes a dephosphorylation step [21, 27], and therefore BLAST searches may retrieve genes encoding unrelated phosphatases. Interestingly, we identified hits for CobT and CobC, but not CobS, in *A. anophagefferens*, which can use pseudocobalamin with DMB, but only with very high levels of the latter (10 μM; Figure 4E). Transcript sequences for *P. lutheri* are available via the Marine Microbial Eukaryote Transcriptome Sequencing Project (MMETSP), a database of 396 unique strains representing ecologically significant and taxonomically diverse marine microbial eukaryotes [35]. This alga, which can use pseudocobalamin alongside DMB, also expresses *COBT*, *COBS*, and *COBC* (Data S2A). Thus, the presence of these novel proteins correlates with the ability to remodel pseudocobalamin, implicating them in B_{12} metabolism. We also identified another 46 candidate remodelers in the MMETSP (Data S2A), including several that encoded *METE*, and so are like *C. reinhardtii* in being independent of a source of B_{12} for growth. In total, the potential remodelers represented ~11% of unique strains and included representatives of the higher class levels Alveolata, Stramenopila, Hacrobia, and Viridiplantae (Data S2B). Incidentally, none of the sequenced *Synechococcus* genomes encode CobT (Data S1), which might explain why *Synechococcus* strains cannot remodel pseudocobalamin to cobalamin in the presence of DMB (Figure 1B).

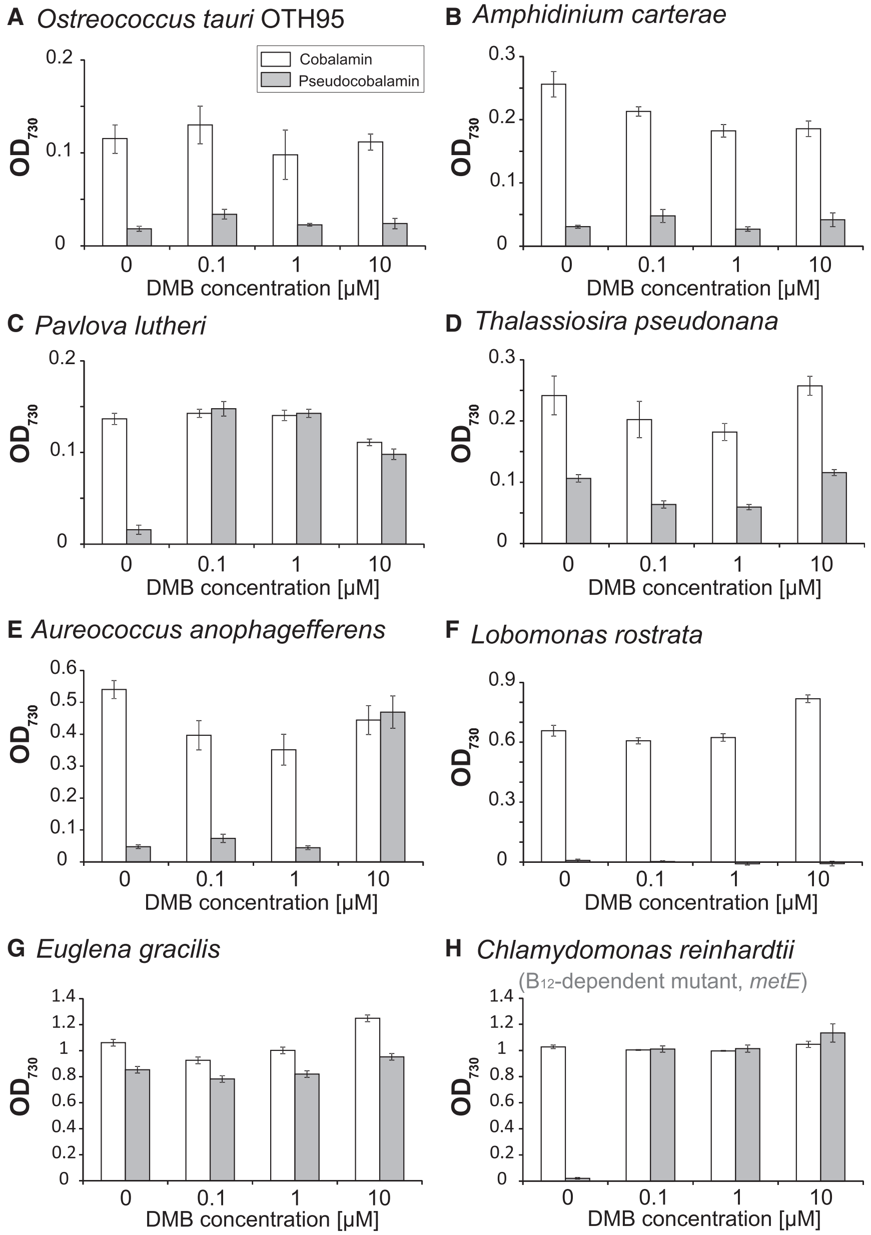

Figure 4. Provision of Lower Ligand Substrate DMB Together with Pseudocobalamin Can Support Growth of Certain B_{12}-Dependent Algae

(A–H) Species were grown in liquid medium (Table S2) without or with 0.7 nM cobalamin (open bars) or 0.7 nM pseudocobalamin (shaded bars) in the presence of different DMB concentrations in batch culture over several transfers or until the cells had died in the $-B_{12}$ treatment. (A) *O. tauri* (OTH95), (B) *A. carteri*, (C) *P. lutheri*, (D) *T. pseudonana*, (E) *A. anophagefferens*, (F) *L. rostrata*, (G) *E. gracilis*, and (H) *C. reinhardtii,* B_{12}-dependent evolved (*metE*) mutant lines are shown [29]. Optical density (OD_{730}) was used to quantify growth (mean ± SEM; n = 3).

DISCUSSION

Eukaryotic microalgae and cyanobacteria are the major components of the phytoplankton in marine and freshwater systems. Because they both inhabit the photic zone, they will compete for resources including light and limiting nutrients such as nitrogen and Fe. We have demonstrated that, in contrast to heterotrophic bacteria such as *D. shibae* (and certain rhizobial bacteria) [28] that make cobalamin, members of the ubiquitous marine *Synechococcus* genus synthesize only pseudocobalamin, in which the lower base is adenine instead of DMB (Figure 1). Moreover, a survey of diverse cyanobacterial genomes, encompassing marine and freshwater species, showed the vast majority do not encode *bluB* or the *bzaABCDE* operon (Data S1) [23, 24]. This strongly suggests that pseudocobalamin is the major form of B_{12} synthesized by most if not all cyanobacteria.

We found that pseudocobalamin is considerably less bioavailable than cobalamin to several B_{12}-dependent algae (Figure 2). This reduced bioavailability suggests these organisms are compromised in their ability to acquire or use pseudocobalamin as a cofactor. Human intrinsic factor, part of the B_{12} uptake system in the gut, exhibits a 500-fold-lower binding affinity for pseudocobalamin [14], thus reducing the bioavailability of the compound to humans. In algae, currently only one protein has been implicated in B_{12} uptake [31] (CBA1), although the precise molecular mechanism and role of CBA1 in B_{12} binding are not fully understood. Nevertheless, the ability of pseudocobalamin to affect the expression of algal B_{12}-responsive genes (Figure 3) and protein levels (Figure S5) suggests this compound can enter algal cells, albeit that it has the opposite effect on *CBA1* to cobalamin, suggesting that the cells are experiencing cobalamin deficiency. Transport of pseudocobalamin into the cell is also indicated by our observed remodeling of pseudocobalamin in *C. reinhardtii* and *P. lutheri* following DMB addition (Figures 4 and 5). The identification of genes encoding enzymes of lower ligand activation (COBT) and nucleotide-loop assembly (COBS) [27] in these algae (Table S1) provides a likely mechanism for corrinoid remodeling. We found no evidence of secretory peptide signals in *C. reinhardtii* COBT or COBS using the green algal subcellular localization tool "PredAlgo" [36], implying that remodeling takes place within the cell and providing further support for the ability of pseudocobalamin to be taken up. Whether these genes have been acquired through lateral gene transfer from a bacterial source, which is thought to be the case for *E. coli* [37], remains unknown. However, of the non-algal sequences retrieved via a BLAST search of the

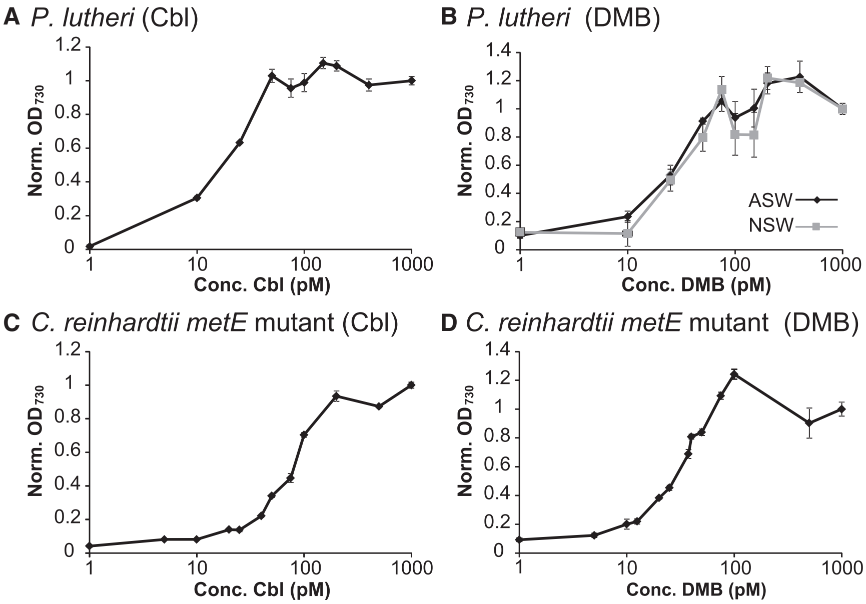

Figure 5. Certain Algae Can Remodel the Lower Axial Ligand of Pseudocobalamin with Exogenously Supplied DMB

(A and B) Relative growth yield of (A) *P. lutheri* cells supplemented with different concentrations of cobalamin after 19 days (values of OD_{730} were normalized as a proportion of growth at 1,000 pM B_{12}) or (B) *P. lutheri* cells supplemented with different concentrations of DMB in the presence of 0.7 nM pseudocobalamin (after 19 days) in artificial seawater or natural filtered seawater (values of OD_{730} normalized to growth at 1,000 pM DMB).

(C and D) Equivalent experiments with *C. reinhardtii*, evolved B_{12}-dependent *metE* mutant [29] are displayed in (C) and (D) after 96 hr growth (mean ± SEM; n = 3).

NCBI non-redundant database with the *C. reinhardtii COBT/COBS*, top hits were derived from the amphipod-associated protist species *Aphanomyces astaci* (4e−57) and *Sphaeroforma arctica* (7e−77), respectively. A broader phylogenetic analysis of *COB* genes will be integral to further understanding of what, at a first glance, appears to be an intriguing evolutionary history. Because algae rely on B_{12} for METH [5], the function of pseudocobalamin as a cofactor for this enzyme is also an important question. Structural data available for the B_{12}-binding pocket and the active site of METH [38, 39] implicate several amino acids in B_{12} binding, with the DMB "tail" buried within a cleft of the active site [40]. Because pseudocobalamin contains an alternative lower base to B_{12}, it seems plausible that algal METH proteins may have reduced binding affinity for pseudocobalamin.

The combination of DMB with pseudocobalamin improves the bioavailability to certain algae. We infer from this that these remodeling algae are able to generate cobalamin from pseudocobalamin + DMB, although we were unable to measure detectable levels of any form of B_{12} in *C. reinhardtii* cells grown under these conditions. It is possible therefore that another form of the vitamin is being generated, though we deem this unlikely. In any case, our results highlight the importance of considering environmental concentrations of DMB. A bioassay to measure free DMB concentrations was recently reported [41]. Analysis of samples derived from host-associated (termite/rumen) and

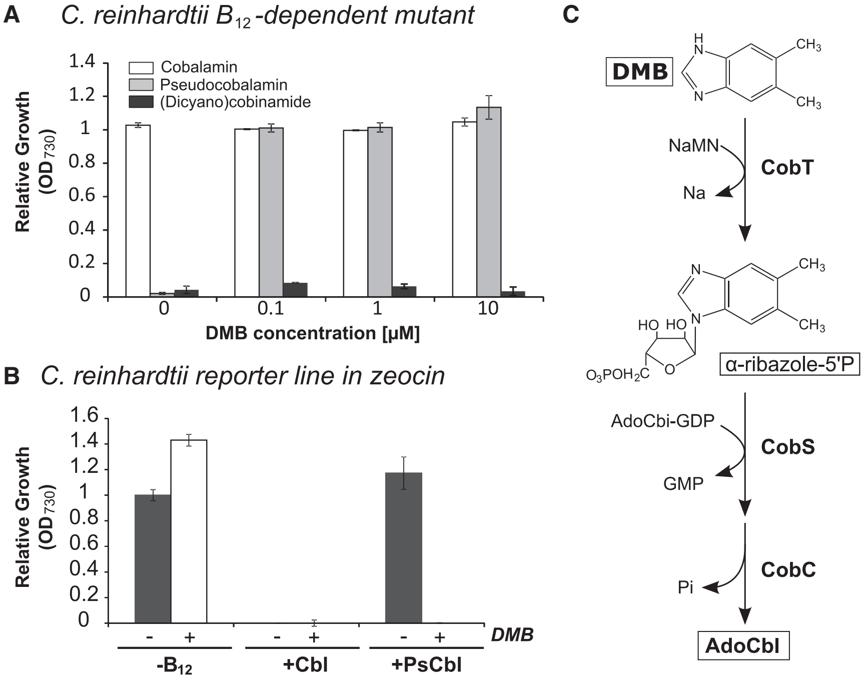

Figure 6. Further Characterization of the Remodeling Phenotype

(A) Relative growth yield (OD_{730}) of *C. reinhardtii* B_{12}-dependent mutant grown with DMB alongside (dicyano)cobinamide, a B_{12} precursor that lacks the DMB ribonucleotide tail. Cells were grown in liquid medium (Table S2) with 0.7 nM cobalamin (open bars), 0.7 nM pseudocobalamin (gray bars), or 0.7 nM (dicyano)cobinamide (black bars) in the presence of different DMB concentrations.

(B) Relative growth yield (OD_{730}) of *C. reinhardtii* reporter line containing a Zeocin resistance gene controlled by the *METE* promoter [32] after 13 days in the presence (white bar) or absence (black bar) of DMB (1 μM) and 20 μg/ml Zeocin without or with 0.7 nM cobalamin or pseudocobalamin. Values of OD_{730} were normalized as a proportion of growth with no B_{12} (mean ± SEM; n = 3).

(C) The pathway for the activation of DMB and nucleotide loop assembly in *S. enterica* (adapted from [27]). CobT catalyzes the attachment of a phosphoribose moiety derived from nicotinate mononucleotide to form α-ribazole phosphate. CobS and CobC catalyze the attachment of the activated base to the cobamide precursor (GDP-cobinamide).

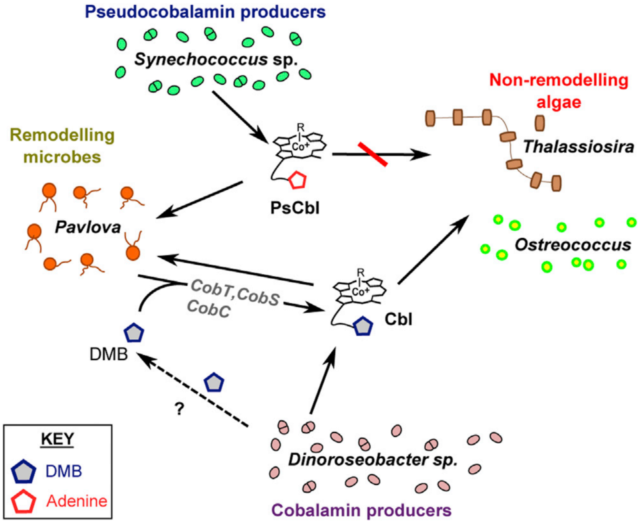

Figure 7. Complex B_{12} Cycling in a Hypothetical Marine Microbial Community

Cobalamin produced by heterotrophic bacteria such as *Dinoroseobacter* sp. is directly usable by algal B_{12} auxotrophs representing major marine taxa, whereas cyanobacterially derived pseudocobalamin is not. However, those algae like *P. lutheri* capable of remodelling can access this essential cofactor if DMB is also present.

natural environmental samples (*Eucalyptus* grove soil/creek water) determined concentrations in the picomolar and sub-picomolar range. Our observation that *P. lutheri* (whose growth can be supported by pseudocobalamin + DMB) could not grow in natural seawater from the English Channel supplemented with pseudocobalamin (Figure 5B) suggests DMB levels were not sufficient in these coastal waters to support remodeling. Nevertheless, further work is required to quantify DMB in marine (and freshwater) environments more broadly. In a similar vein, a recent field study by Heal et al. [42] quantified the relative abundance of four upper axial variants of B_{12} (CN, Me, Ado, and OH) in estuarine waters of the Puget Sound, but levels of lower axial ligand variants are unknown.

Representatives of all major algal lineages require B_{12}, yet its biosynthesis is limited to a subset of prokaryotes. As such, the flux of B_{12} between microbes is integral to the growth of auxotrophic species. Our results imply heterotrophic bacteria are likely to be a more important source of B_{12} for eukaryotic algae than cyanobacteria. An increasing body of evidence provided by this study and others [33, 34, 43, 44] suggests the relationship between requirers and providers has become blurred by the existence of scavengers and remodelers. That different B_{12} forms are not functionally equivalent between organisms means that biochemical transformations between vitamer classes are essential for this micronutrient to reach different members of the community. This complicates our current view of B_{12} cycling in aquatic environments (Figure 7). Whether cyanobacteria synthesize a currency of B_{12} that is inaccessible to competing eukaryotic microbes as a strategy to exclude competitors remains unknown. Nevertheless, the observation that certain algae possess a counter-mechanism to convert pseudocobalamin to a bioavailable form suggests the selective pressure to devise and refine strategies of B_{12} acquisition/utilization in order to enhance accessibility to this limiting micronutrient is strong. In any case, the importance of B_{12} and its derivatives in structuring microbial communities in aquatic ecosystems may have been previously underestimated.

EXPERIMENTAL PROCEDURES

Bioinformatics Approaches

A full description of sequence similarity search parameters is provided in the Supplemental Experimental Procedures.

Chemicals

Upper axial cyano forms of cobalamin/pseudocobalamin were used for all B_{12}-amendment experiments. Cyanocobalamin was purchased from Sigma-Aldrich UK. Cyanopseudocobalamin was prepared by guided biosynthesis from a culture of *Propionibacterium acidi-propionici* DSM 20273 as described previously and confirmed by UV-Vis, circular dichroism (CD), mass, and nuclear magnetic resonance (NMR) spectroscopic analysis [12].

Strains and Growth Conditions

Details of microbial strains and culture conditions are provided in the Supplemental Experimental Procedures and Table S2.

Molecular Methods

RNA Extraction and qRT-PCR

Total RNA was extracted [6] and treated with the Ambion Turbo DNase-Free Kit to remove genomic DNA. RNA was reverse transcribed into cDNA with SuperScript II (Invitrogen). Details of qRT-PCR are given in the Supplemental Experimental Procedures and Table S3.

Western Blotting

Total protein was extracted and western blot experiments performed as described in [32].

ACCESSION NUMBERS

The data described in this manuscript are available at https://www.repository.cam.ac.uk/handle/1810/254413.

SUPPLEMENTAL INFORMATION

Supplemental Information includes Supplemental Experimental Procedures, five figures, three tables, and two datasets and can be found with this article online at http://dx.doi.org/10.1016/j.cub.2016.02.041.

AUTHOR CONTRIBUTIONS

K.E.H., A.D.L., A.H., U.J.K., S.S., D.J.S., M.J.W., and A.G.S. designed the research; K.E.H., A.D.L., A.H., U.J.K., and S.S. performed the experiments; K.E.H., A.D.L., A.H., U.J.K., M.J.W., and A.G.S. analyzed the data; and K.E.H., D.J.S., B.K., M.J.W., and A.G.S. wrote the paper.

ACKNOWLEDGMENTS

We thank G. Wheeler (MBA, UK) for providing L4 seawater. We also thank M. Croft, S. Hodson, M. Scaife, L. Norman, and E. Kazamia for technical support and helpful insight. Funding was from the BBSRC (BB/I013164/1 to K.E.H. and A.G.S. and BB/K009249/1 to A.D.L. and M.J.W.), EU FP7 Marie Curie ITN Photo.Comm, no. 317184 (to A.G.S. and U.J.K.), The Swiss National Science Foundation (grant nos. PBEZA–115703 and PA00P3–124169 to S.S.), and the EU project MaCuMBA (no. 311975 to D.J.S.).

Received: January 4, 2016
Revised: February 15, 2016
Accepted: February 17, 2016
Published: March 31, 2016

REFERENCES

1. Field, C.B., Behrenfeld, M.J., Randerson, J.T., and Falkowski, P. (1998). Primary production of the biosphere: integrating terrestrial and oceanic components. Science *281*, 237–240.
2. Falkowski, P.G., Barber, R.T., and Smetacek, V. (1998). Biogeochemical controls and feedbacks on ocean primary production. Science *281*, 200–207.
3. Croft, M.T., Warren, M.J., and Smith, A.G. (2006). Algae need their vitamins. Eukaryot. Cell *5*, 1175–1183.
4. Fenech, M. (2012). Folate (vitamin B_9) and vitamin B_{12} and their function in the maintenance of nuclear and mitochondrial genome integrity. Mutat. Res. *733*, 21–33.
5. Croft, M.T., Lawrence, A.D., Raux-Deery, E., Warren, M.J., and Smith, A.G. (2005). Algae acquire vitamin B_{12} through a symbiotic relationship with bacteria. Nature *438*, 90–93.
6. Helliwell, K.E., Wheeler, G.L., Leptos, K.C., Goldstein, R.E., and Smith, A.G. (2011). Insights into the evolution of vitamin B_{12} auxotrophy from sequenced algal genomes. Mol. Biol. Evol. *28*, 2921–2933.
7. Kurata, A. (1986). Chrysophytes: Aspects and Problems, J.A. Kristiansen, ed. (Cambridge University Press), pp. 185–196.
8. Sañudo-Wilhelmy, S.A., Cutter, L.S., Durazo, R., Smail, E.A., Gómez-Consarnau, L., Webb, E.A., Prokopenko, M.G., Berelson, W.M., and Karl, D.M. (2012). Multiple B-vitamin depletion in large areas of the coastal ocean. Proc. Natl. Acad. Sci. USA *109*, 14041–14045.
9. Sañudo-Wilhelmy, S.A., Gobler, C.J., Okbamichael, M., and Taylor, G.T. (2006). Regulation of phytoplankton dynamics by vitamin B_{12}. Geophys. Res. Lett. *33*, L04604.
10. Wagner-Döbler, I., Ballhausen, B., Berger, M., Brinkhoff, T., Buchholz, I., Bunk, B., Cypionka, H., Daniel, R., Drepper, T., Gerdts, G., et al. (2010). The complete genome sequence of the algal symbiont *Dinoroseobacter shibae*: a hitchhiker's guide to life in the sea. ISME J. *4*, 61–77.
11. Banerjee, R., and Ragsdale, S.W. (2003). The many faces of vitamin B_{12}: catalysis by cobalamin-dependent enzymes. Annu. Rev. Biochem. *72*, 209–247.
12. Hoffmann, B., Oberhuber, M., Stupperich, E., Bothe, H., Buckel, W., Konrat, R., and Kräutler, B. (2000). Native corrinoids from *Clostridium cochlearium* are adeninylcobamides: spectroscopic analysis and identification of pseudovitamin B_{12} and factor A. J. Bacteriol. *182*, 4773–4782.
13. Stupperich, E., and Kräutler, B. (1988). Pseudo vitamin B_{12} or 5-hydroxybenzimidazolyl-cobamide are the corrinoids found in methanogenic bacteria. Arch. Microbiol. *149*, 268–271.
14. Stupperich, E., and Nexø, E. (1991). Effect of the cobalt-N coordination on the cobamide recognition by the human vitamin B_{12} binding proteins intrinsic factor, transcobalamin and haptocorrin. Eur. J. Biochem. *199*, 299–303.
15. Watanabe, F., Katsura, H., Takenaka, S., Fujita, T., Abe, K., Tamura, Y., Nakatsuka, T., and Nakano, Y. (1999). Pseudovitamin B_{12} is the predominant cobamide of an algal health food, spirulina tablets. J. Agric. Food Chem. *47*, 4736–4741.
16. Miyamoto, E., Tanioka, Y., Nakao, T., Barla, F., Inui, H., Fujita, T., Watanabe, F., and Nakano, Y. (2006). Purification and characterization of a corrinoid-compound in an edible cyanobacterium *Aphanizomenon flos-aquae* as a nutritional supplementary food. J. Agric. Food Chem. *54*, 9604–9607.
17. Flombaum, P., Gallegos, J.L., Gordillo, R.A., Rincón, J., Zabala, L.L., Jiao, N., Karl, D.M., Li, W.K., Lomas, M.W., Veneziano, D., et al. (2013). Present and future global distributions of the marine Cyanobacteria *Prochlorococcus* and *Synechococcus*. Proc. Natl. Acad. Sci. USA *110*, 9824–9829.
18. Bonnet, S., Webb, E.A., Panzeca, C., Karl, D.M., Capone, D.G., and Sañudo-Wilhelmy, S.A. (2010). Vitamin B_{12} excretion by cultures of the marine cyanobacteria *Crocosphaera* and *Synechococcus*. Limnol. Oceanogr. *55*, 1959–1964.
19. Droop, M.R. (1957). Auxotrophy and organic compounds in the nutrition of marine phytoplankton. J. Gen. Microbiol. *16*, 286–293.
20. Scanlan, D.J., Ostrowski, M., Mazard, S., Dufresne, A., Garczarek, L., Hess, W.R., Post, A.F., Hagemann, M., Paulsen, I., and Partensky, F. (2009). Ecological genomics of marine picocyanobacteria. Microbiol. Mol. Biol. Rev. *73*, 249–299.
21. Warren, M.J., Raux, E., Schubert, H.L., and Escalante-Semerena, J.C. (2002). The biosynthesis of adenosylcobalamin (vitamin B_{12}). Nat. Prod. Rep. *19*, 390–412.
22. Sañudo-Wilhelmy, S.A., Gómez-Consarnau, L., Suffridge, C., and Webb, E.A. (2014). The role of B vitamins in marine biogeochemistry. Annu. Rev. Mar. Sci. *6*, 339–367.
23. Taga, M.E., Larsen, N.A., Howard-Jones, A.R., Walsh, C.T., and Walker, G.C. (2007). BluB cannibalizes flavin to form the lower ligand of vitamin B_{12}. Nature *446*, 449–453.
24. Hazra, A.B., Han, A.W., Mehta, A.P., Mok, K.C., Osadchiy, V., Begley, T.P., and Taga, M.E. (2015). Anaerobic biosynthesis of the lower ligand of vitamin B_{12}. Proc. Natl. Acad. Sci. USA *112*, 10792–10797.
25. Kazamia, E., Czesnick, H., Nguyen, T.T., Croft, M.T., Sherwood, E., Sasso, S., Hodson, S.J., Warren, M.J., and Smith, A.G. (2012). Mutualistic interactions between vitamin B_{12} -dependent algae and heterotrophic bacteria exhibit regulation. Environ. Microbiol. *14*, 1466–1476.
26. Hartmann, M., Gomez-Pereira, P., Grob, C., Ostrowski, M., Scanlan, D.J., and Zubkov, M.V. (2014). Efficient CO_2 fixation by surface *Prochlorococcus* in the Atlantic Ocean. ISME J. *8*, 2280–2289.
27. Anderson, P.J., Lango, J., Carkeet, C., Britten, A., Kräutler, B., Hammock, B.D., and Roth, J.R. (2008). One pathway can incorporate either adenine or dimethylbenzimidazole as an alpha-axial ligand of B_{12} cofactors in *Salmonella enterica*. J. Bacteriol. *190*, 1160–1171.
28. Campbell, G.R.O., Taga, M.E., Mistry, K., Lloret, J., Anderson, P.J., Roth, J.R., and Walker, G.C. (2006). *Sinorhizobium meliloti* bluB is necessary for production of 5,6-dimethylbenzimidazole, the lower ligand of B_{12}. Proc. Natl. Acad. Sci. USA *103*, 4634–4639.
29. Helliwell, K.E., Collins, S., Kazamia, E., Purton, S., Wheeler, G.L., and Smith, A.G. (2015). Fundamental shift in vitamin B_{12} eco-physiology of a model alga demonstrated by experimental evolution. ISME J. *9*, 1446–1455.
30. Torrents, E., Trevisiol, C., Rotte, C., Hellman, U., Martin, W., and Reichard, P. (2006). *Euglena gracilis* ribonucleotide reductase: the eukaryote class II enzyme and the possible antiquity of eukaryote B_{12} dependence. J. Biol. Chem. *281*, 5604–5611.
31. Bertrand, E.M., Allen, A.E., Dupont, C.L., Norden-Krichmar, T.M., Bai, J., Valas, R.E., and Saito, M.A. (2012). Influence of cobalamin scarcity on diatom molecular physiology and identification of a cobalamin acquisition protein. Proc. Natl. Acad. Sci. USA *109*, E1762–E1771.
32. Helliwell, K.E., Scaife, M.A., Sasso, S., Araujo, A.P.U., Purton, S., and Smith, A.G. (2014). Unraveling vitamin B_{12}-responsive gene regulation in algae. Plant Physiol. *165*, 388–397.
33. Gray, M.J., and Escalante-Semerena, J.C. (2009). The cobinamide amidohydrolase (cobyric acid-forming) CbiZ enzyme: a critical activity of the cobamide remodelling system of *Rhodobacter sphaeroides*. Mol. Microbiol. *74*, 1198–1210.
34. Yi, S., Seth, E.C., Men, Y.J., Stabler, S.P., Allen, R.H., Alvarez-Cohen, L., and Taga, M.E. (2012). Versatility in corrinoid salvaging and remodeling pathways supports corrinoid-dependent metabolism in *Dehalococcoides mccartyi*. Appl. Environ. Microbiol. *78*, 7745–7752.
35. Keeling, P.J., Burki, F., Wilcox, H.M., Allam, B., Allen, E.E., Amaral-Zettler, L.A., Armbrust, E.V., Archibald, J.M., Bharti, A.K., Bell, C.J., et al. (2014). The Marine Microbial Eukaryote Transcriptome Sequencing Project (MMETSP): illuminating the functional diversity of eukaryotic life in the oceans through transcriptome sequencing. PLoS Biol. *12*, e1001889.
36. Tardif, M., Atteia, A., Specht, M., Cogne, G., Rolland, N., Brugière, S., Hippler, M., Ferro, M., Bruley, C., Peltier, G., et al. (2012). PredAlgo: a

new subcellular localization prediction tool dedicated to green algae. Mol. Biol. Evol. *29*, 3625–3639.

37. Lawrence, J.G., and Roth, J.R. (1995). The cobalamin (coenzyme B_{12}) biosynthetic genes of *Escherichia coli*. J. Bacteriol. *177*, 6371–6380.

38. Drennan, C.L., Huang, S., Drummond, J.T., Matthews, R.G., and Lidwig, M.L. (1994). How a protein binds B_{12}: A 3.0 Å X-ray structure of B_{12}-binding domains of methionine synthase. Science *266*, 1669–1674.

39. Ludwig, M.L., and Matthews, R.G. (1997). Structure-based perspectives on B_{12}-dependent enzymes. Annu. Rev. Biochem. *66*, 269–313.

40. Tollinger, M., Konrat, R., Hilbert, B.H., Marsh, E.N.G., and Kräutler, B. (1998). How a protein prepares for B_{12} binding: structure and dynamics of the B_{12}-binding subunit of glutamate mutase from *Clostridium tetanomorphum*. Structure *6*, 1021–1033.

41. Crofts, T.S., Men, Y., Alvarez-Cohen, L., and Taga, M.E. (2014). A bioassay for the detection of benzimidazoles reveals their presence in a range of environmental samples. Front Microbiol *5*, 592.

42. Heal, K.R., Carlson, L.T., Devol, A.H., Armbrust, E.V., Moffett, J.W., Stahl, D.A., and Ingalls, A.E. (2014). Determination of four forms of vitamin B_{12} and other B vitamins in seawater by liquid chromatography/tandem mass spectrometry. Rapid Commun. Mass Spectrom. *28*, 2398–2404.

43. Men, Y., Seth, E.C., Yi, S., Crofts, T.S., Allen, R.H., Taga, M.E., and Alvarez-Cohen, L. (2015). Identification of specific corrinoids reveals corrinoid modification in dechlorinating microbial communities. Environ. Microbiol. *17*, 4873–4884.

44. Crofts, T.S., Seth, E.C., Hazra, A.B., and Taga, M.E. (2013). Cobamide structure depends on both lower ligand availability and CobT substrate specificity. Chem. Biol. *20*, 1265–1274.

Current Biology, Volume *26*

Supplemental Information

Cyanobacteria and Eukaryotic Algae Use Different Chemical Variants of Vitamin B_{12}

Katherine Emma Helliwell, Andrew David Lawrence, Andre Holzer, Ulrich Johan Kudahl, Severin Sasso, Bernhard Kräutler, David John Scanlan, Martin James Warren, and Alison Gail Smith

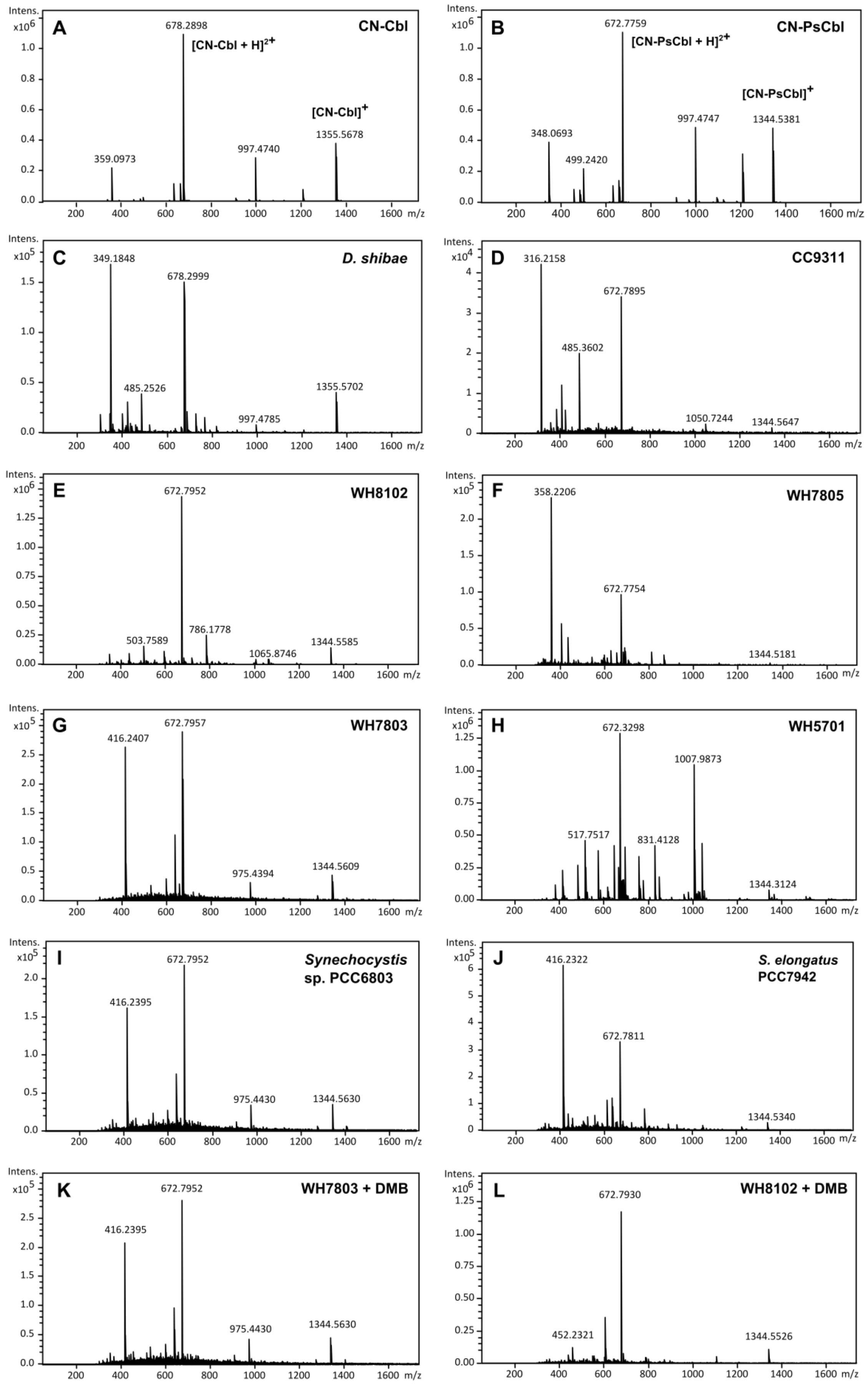

A
CN-Cbl
678.2898
[CN-Cbl + H]2+
[CN-Cbl]+
359.0973
997.4740
1355.5678
B
CN-PsCbl
672.7759
[CN-PsCbl + H]2+
[CN-PsCbl]+
348.0693
499.2420
997.4747
1344.5381
C
D. shibae
349.1848
678.2999
485.2526
997.4785
1355.5702
D
CC9311
316.2158
672.7895
485.3602
1050.7244
1344.5647
E
WH8102
672.7952
503.7589
786.1778
1065.8746
1344.5585
F
WH7805
358.2206
672.7754
1344.5181
G
WH7803
416.2407
672.7957
975.4394
1344.5609
H
WH5701
672.3298
1007.9873
517.7517
831.4128
1344.3124
I
Synechocystis sp. PCC6803
672.7952
416.2395
975.4430
1344.5630
J
S. elongatus PCC7942
416.2322
672.7811
1344.5340
K
WH7803 + DMB
672.7952
416.2395
975.4430
1344.5630
L
WH8102 + DMB
672.7930
452.2321
1344.5526
Intens.
m/z

Figure S1 ESI$^+$ mass spectra of B_{12} standards and B_{12} extracted from different bacteria. Related to Figure 1. **A.** cobalamin**;** **B.** pseudocobalamin; **C.** Alpha-proteobacterium *D. shibae*; **D.** *Synechococcus* sp. CC9311; **E.** *Synechococcus* sp. WH8102; **F.** *Synechococcus* sp. WH7805; **G.** *Synechococcus* sp. WH7803; **H**. *Synechococcus* sp. WH5701**;** **I.** *Synechocystis* sp. PCC6803*;* **J.** *Synechococcus elongatus* PCC7942; **K.** and **L.** *Synechococcus* sp. WH7803 and WH8103 respectively, grown with 1 µM DMB. Data from all cyanobacterial samples contain peaks which are characteristic of CN-pseudocobalamin with m/z 1344.5 $[CN\text{-}PsCbl]^+$ and m/z 672.7 $[CN\text{-}PsCbl + H]^{2+}$ but not m/z 1355.5 $[CN\text{-}Cbl]^+$ and m/z 678.3 $[CN\text{-}Cbl + H]^{2+}$ for CN-cobalamin. In contrast, *D. shibae* has the characteristic cobalamin ions.

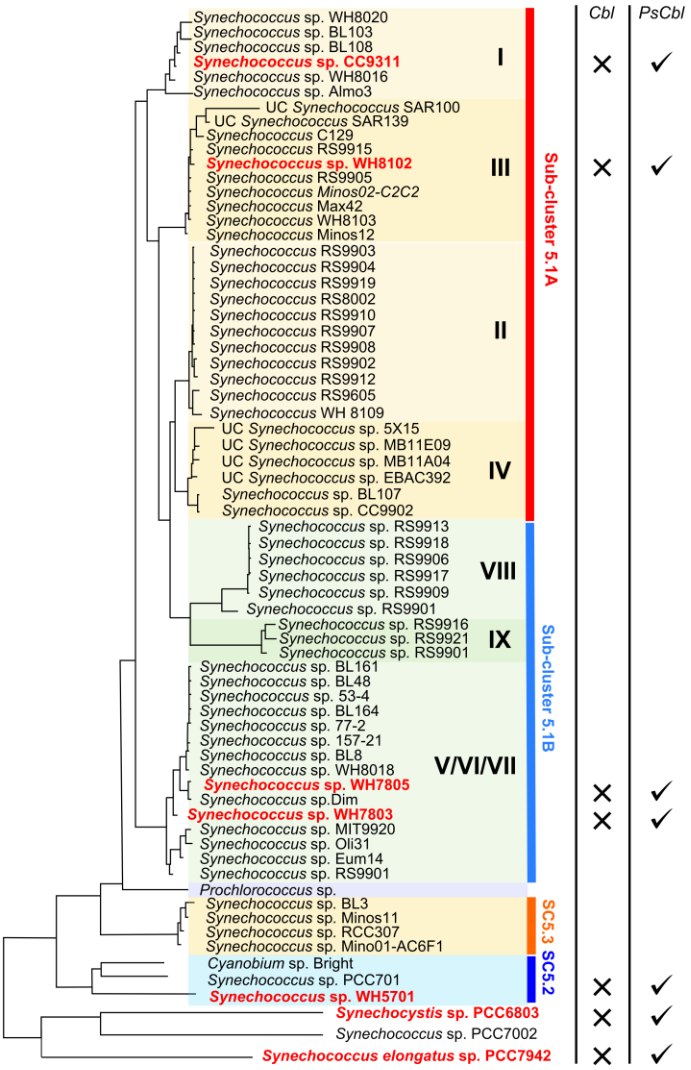

Figure S2 *Synechococcus* strains synthesise pseudocobalamin rather than cobalamin. Related to Figure 1. *Synechococcus* phylogeny adapted from [S1] indicating in red the strains surveyed in this study. Key: Cbl = synthesises cobalamin; PsCbl = synthesised pseudocobalamin.

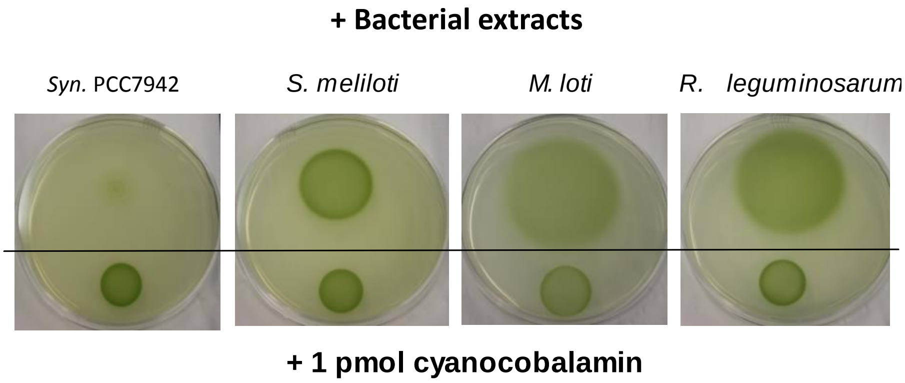

Figure S3 Growth of *L. rostrata* is supported by extracts of rhizobia but not cyanobacteria. Related to Figure 2. A lawn of *L. rostrata* cells was plated in top agar onto TAP solid medium, then 10 µl of different B_{12}-containing solutions i.e. freeze-dried extracts from different bacteria, each containing approximately 10 pmol of B_{12} (quantified using the *S. typhimurium* bioassay [S2]), were spotted onto the surface. The plates were left in the light for 10 days and then photographed. The cell extract from *Synechococcus elongatus* PCC7942 does not support growth, whereas a distinct lawn of algal cells is visible for each of the three rhizobial species *Sinorhizobium meliloti* 1021, *Mesorhizoium loti* MAFF303099 and *Rhizobium leguminosarum* 3841. The lower spot on each plate is from the addition of 1 pmol cobalamin standard.

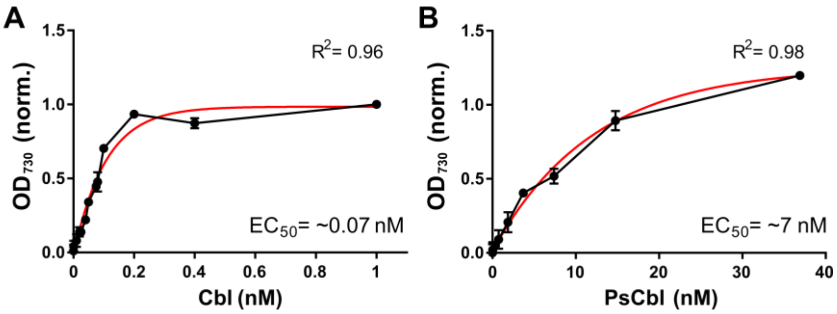

Figure S4 Dose response of *C. reinhardtii* B_{12}-dependent *metE* mutant [S3] with different B_{12} variants. Related to Figure 2. **A.** Cells were grown in TAP + Cbl for 96 hours and growth measured as OD_{730} incubated. **B**. As for (A) but cells were supplemented with PsCbl. OD_{730} was normalized to OD_{730} on 0.7 nM Cbl. Nonlinear fits were applied to the data and nutrient concentrations supporting half maximal biomass accumulation were estimated (EC_{50}) (mean ± SEM, *n*=3).

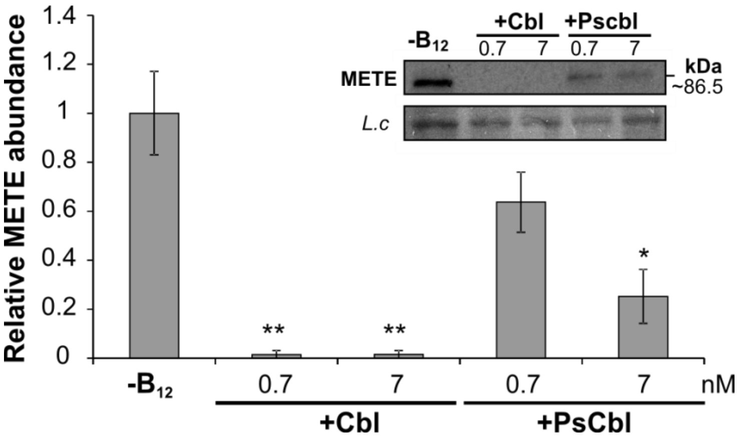

Figure S5. Western blot analysis of METE protein in *C. reinhardtii*. Related to Figure 3. Cells were grown with no supplementation or with 0.7 or 7 nM of Cbl or PsCbl, and total soluble protein extracted after 48 hours. Four µg of total protein was loaded for each sample. After transfer of protein to PVDF membrane and staining with Ponceau S, the blots were probed with a METE antibody [S4]. Band intensity analysis (integrated density) using ImageJ software [S5] allowed METE abundance to be quantified and normalised to the quantity of loaded protein from the stained blot (L.c: loading control). Mean ± SEM, n=3; *$P \leq 0.05$, **$P \leq 0.01$, ***$P \leq 0.001$ compared to the treatment without B_{12} (two-tailed Student's t test).

Table S1. Analysis of 13 sequenced algal genomes (*and one transcriptome) for proteins known in bacteria to be involved in DMB activation/lower ligand assembly. Related to Figures 4 and 5.

Searches of algal genomes utilised the Joint Genome Institute portal (http://genome.jgi.doe.gov/) or independent sources (*Ectocarpus siliculosus:* http://bioinformatics.psb.ugent.be/genomes/view/Ectocarpus-siliculosus, and *Cyanidioschyzon merolae*, http://merolae.biol.s.u-tokyo.ac.jp/). The data shown from *Pavlova lutheri* (strain name: RCC 1537, or alternatively CCAP 931/1) is from the Marine Microbial Eukaryote Transcriptome Sequencing Project [S6]. E-value and protein ID of retrieved sequences are given. All hits shown were verified via Pfam analysis to contain the expected conserved domains analogous to the relevant query protein used to carry out the BLASTP search (Supplemental Experimental Procedures), namely: nicotinate-nucleotide-DMB-phosphoribosyltransferase domain (CobT), Cobalamin-5-phosphate synthase domain (CobS) and Histidine phosphatase superfamily (branch 1) (CobC). Species shaded grey have been shown experimentally to require B_{12}.

Group	Species (and genome version)	Lower ligand assembly (**Early insertion pathway)**			Remodelling (*R.sphaeroides*)	
		CobT	*CobS*	*CobC*	*CbiZ*	*CbiB*
Rhodophyta	*Cyanidoschyzon merolae*	✘	✘	✓(5e-7; id: CMN085C)	✘	✘
Chlorophyta	*Chlamydomonas reinhardtii* (*v5.5*)	✓(6.7e-10; id: Cre12.g555500.t1.2)	✓(1.4e-5; id: Cre01.g036950.t1.2)	✓(2e-10; id: Cre10.g460300.t1.2)<br>✓(5.9e-9;id: Cre05.g232550.t1.2)<br>✓(2.5e-7; id: Cre13.g564000.t1.2)	✘	✘
	Volvox carteri f. *nagariensis* EVE (*v2*)	✘	✘	✓(6.8e-14; id:Vocar20015114m)	✘	✘
	Micromonas pusilla RCC299 (*v3*)	✘	✘	✓(4.4e-10; id:50990)	✘	✘
	Ostreococcus tauri OTH95(*v2*)	✘	✘	✘	✘	✘
	Coccomyxa sp. C-169 (*v2*)	✘	✘	✓(1.4e-12; id:14103)<br>✓(3.1e-9; id:28443)	✘	✘
	Chlorella variabilis NC64A	✘	✘	✘	✘	✘
Chromalveolata	*Pavlova lutheri* (strain name: RCC 1537, or alternatively CCAP 931/1)	✓<br>(1.4 e-17; 14947)	✓(9.9 e-15;2612)	✓(1.2e-6;1018)	✘	✘
	Emiliania huxleyi (CCMP1516) (*v1*)	✓(1.17e-26; id: 241304)	✓(3e-14; id: 95466)	✓(2.44e-8; id:213567)<br>✓(1.67e-8; id:223722)	✘	✘
	Phaeodactylum tricornutum CCAP1055/1 (*v2*)	✓(2.58e-25; id:16445)	✘	✘	✘	✘
	Fragilariopsis cylindrus CCMP 1102 (v1)	✓(2.22e-31; id:180280)	✘	✘	✘	✘
	Thalassiosira pseudonana (v3)	✘	✘	✓(7.27e-9; id:264296)	✘	✘
	Aureococcus anophagefferens clone 1984 (*v1*)	✓(3.12e-32; id:58669)	✘	✓(6.14e-0; id:71905)<br>✓(1.85e-6; id:14569)	✘	✘
	Ectocarpus siliculosus	✘	✘	✓(3e-11; id: Esi0119_0093)	✘	✘

Table S2: Strains, origin, and growth conditions for each alga grown during this study. Related to Figures 2, 4, 5 and 6.

Species	Media	Conditions	Origin
Algae			
Amphidinium carterae (CCAP 1102/6)	F/2[1]	25°C, standing, continuous light (20 μmol/m^2/sec)	A gift from Professor Christopher Howe, University of Cambridge, UK
Aureococcus anophagefferens (CCMP 1984)	L1[2]	18°C, standing, 16 h:8 h light:dark (20 μmol/m^2/sec)	Roscoff Culture Collection, France
Chlamydomonas reinhardtii (WT st.12) a wild-type strain derived from strain 137c)	TAP3	25°C, shaking 100 r.p.m, continuous light (120 μmol/m^2/sec)	A gift from Dr Saul Purton, UCL, London, UK
Chlamydomonas reinhardtii evolved B_{12}-dependent mutant (*metE*$^-$) (20) (in WT st.12 background)	TAP	25°C, shaking 100 r.p.m, continuous light (120 μmol/m^2/sec)	[S3]
Euglena gracilis	TAP	25°C, shaking, continuous light (100 μmol/m^2/sec)	A gift from Prof Alan Battersby, University of Cambridge, UK
Lobomonas rostrata (SAG 45/2)	TAP	25°C, shaking 100 r.p.m, continuous light (120 nmol/m^2/sec)	Experimental Phycology and Culture Collection of Algae at the University of Goettingen (EPSAG), Germany
Pavlova lutheri (strain name: RCC 1537, or alternatively CCAP 931/1)	F/2	25°C, standing, continuous light (20 μmol/m^2/sec)	A gift from Dr Craig Pooley, Swansea University, UK.
Ostreococcus tauri (OTH95)	L1	18°C, standing, 16 h:8 h light:dark (20 μmol/m^2/sec)	A gift from Dr Herve Moreau Oceanological Observatory of Banyuls-ser-Mer, France
Thalassiosira pseudonana (CCMP 1335)	F/2 + Si	18°C, standing, 16 h:8 h light:dark (20 μmol/m^2/sec)	A gift from Dr Richard Dorrell, Ecole Normale Supérieure, Paris, France
Phaeodactylum tricornutum (CCAP 1052/B)	F/2 + Si	25°C, shaking 120 r.p.m, continuous light (20 μmol/m^2/sec)	CCAP, Oban, UK
Cyanobacteria			
Synechococcus sp. WH8102, WH7803, WH7805, WH5701	ASW[4]	24°C, standing, continuous light (5 μmol/m^2/sec)	University of Warwick, UK
Synechococcus sp. CC9311	ASW +	24°C, standing, continuous light (5	University of Warwick, UK

	L1 trace metals	µmol/m^2/sec)	
Synechocystis sp. PCC 6803	BG11[5]	25°C, shaking 100 r.p.m, continuous light (100 µmol/m^2/sec)	CCAP, Oban, UK
Synechococcus elongatus PCC7942	BG11	25°C, shaking 100 r.p.m, continuous light (100 µmol/m^2/sec)	A gift from Dr David Lea-Smith, University of Cambridge, UK.

[1]f/2 [S7, S8]
[2]L1 [S9]

[3]TAP = Tris-Acetate-Phosphate medium (i.e. heterotrophic) [S10]
[4] ASW [S11]

[5] BG-11 [S12]

Table S3. Primers for qPCR analysis used in this study. Related to Figure 3.

Gene	Primer name	Sequence (5'→3')
C. reinhardtii		
METE	C.r_METE_qPCR_F5	GTTCCCCACCACAACCATC
	C.r_METE_qPCR_R5	CGATGAGGCCCTCGTACTC
SAHH	C.r SAHH_F1-	ACAACGCCGAGTTCAAGATT
	C.r SAHH_R1-	CCCATCACCTTCTTGGACAT
ACTIN	C.r ACTIN Exon2_F	CTGGCTTTGCTGGTGATGAT
	C.r ACTIN exon 3 R	CCAGAGTCCAGCACGATACC
UBQ	C.r UBQ2 3UTR F	GCGATTTCTCGTTGGGCAGT
	C.r UBQ2 3UTR R	TGGCCCATCCACTTGTCCTT
RACK1	C.r RCK1 exon 3 F	GCCACACCGAGTGGGTGTCGTGCG
	C.r RCK1 exon 4 R	CCTTGCCGCCCGAGGCGCACAGCG
P. tricornutum		
METE	P.tri METEb F-	TTTTGGATTGAGTCGCTGGGAA
	P.tri METEb R-	TGGTAGTTGCTCGTGATCCATT
CBA1	P.tri_aCBA1_F1	AGTCTTCGATTACCAGGCATCC
	P.tri_aCBA1_R1	TACCAACAACGGCACAAAAGTC
H4	P.tri HISTONE H4-F1	AGGTCCTTCGCGACAATATC
	P.tri HISTONE H4-R1	ACGGAATCACGAATGACGTT
RPS	P.tri RPS-F1	CGAAGTCAACCAGGAAACCAA
	P.tri RPS-R1	GTGCAAGAGACCGGACATACC
TBP	P.tri TBP-F1	ACCGGAGTCAAGAGCACACAC
	P.tri TBP-R1	CGGAATGCGCGTATACCAGT

Supplemental Experimental Procedures

Sequence similarity searches and analysis of putative orthologs

There are two pathways for B_{12} biosynthesis: the 'early cobalt-insertion' (also known as the anaerobic pathway) and 'late cobalt-insertion' (aerobic) pathways. Orthologues of genes encoding $\mathbf{B_{12}}$-synthesis enzymes, including BluB and the *bzaABCDE* operon, were searched for in sequenced cyanobacterial genomes using BLASTP [S13] and a set of template sequences (Dataset S1) as input sequences. The template sequences are derived from UniProt [S14] and have all been functionally verified. We used an e-value cut-off of $<10^{-5}$ and identified the most similar protein sequence for each of the template genes for the investigated species. We then used the rps-BLAST algorithm to identify protein domains for the best matching sequences from the TIGRFAM [S15], Conserved Domain Database [S16], Pfam [S17] and Clusters of Orthologous Groups of proteins [S18] databases. We compared the identified domains with a list of expected domains for each gene (Dataset S1), and used a threshold of at least one shared domain between the expected and identified domains as a requirement for the gene to be classified as a putative B_{12}-synthesis gene. In order to classify each species as either capable of *de novo* B_{12} synthesis or not, we used a threshold requiring that $\geq$ 15 genes from the early cobalt-insertion pathway to be present in a species for it to be classified as a B_{12} producer. At the time of the analysis (27th July, 2015) 123 sequenced cyanobacterial genomes were available in NCBI, representing the orders: Chroococcales, Gloeobacterales, Nostocales, Oscillatoriales, Pleurocapsales, Prochlorales and Stigonematales (Dataset S1).

Searches of algal genomes for orthologues of bacterial *cobT*, *cobS*, *cobC* (Table S1) utilised the Joint Genome Institute portal (http://genome.jgi.doe.gov/) or independent sources (*Ectocarpus siliculosus:* http://bioinformatics.psb.ugent.be/genomes/view/Ectocarpus-siliculosus, and *Cyanidioschyzon merolae,* http://merolae.biol.s.u-tokyo.ac.jp/). The origin and sequence IDs of the proteins that were used to perform BLASTP searches are as follows: CobT (*S. typhimurium*, gi: 1253537), CobS (*S. typhimurium*, gi: 20141415), CobC (*S. typhimurium*, gi: 20141373), CbiZ (*Rhodobacter sphaeroides*, gi: 488807154), CbiB (*S. typhimurium,* gi: 543942). To verify the identity of putative orthologous algal proteins we employed: 1) multiple sequence alignment using Jalview [S19] to check visually each gene model, 2) Pfam (27.0) analysis [S20] to identify conserved functional domains in each protein, including: Nicotinate-nucleotide-DMB-phosphoribosyltransferase domain (CobT), Cobalamin-5-phosphate synthase domain (CobS) and Histidine phosphatase superfamily (branch 1) (CobC), analogous to the relevant query protein used for BLASTP searches. For mining of the MMETSP dataset sequence similarity searches for CobT and CobS were carried out with *Emiliania huxleyi* proteins (protein id: 241304 and 95466, respectively). Subcellular localisation analysis was carried out on *C. reinhardtii* COBT and COBS using PredAlgo [S21] (according to the default settings) [S22].

Strains and growth conditions

The strains, origin, and growth conditions for each alga grown during this study are summarised in Table S2. Details of media used are from the Culture Collection of Algae and Protozoa, Oban, UK (http://www.ccap.ac.uk/). Growth assays were performed in 24-well plates using 2 mL medium supplemented with variable final concentrations of cyanocobalamin, cyanopseudocobalamin, and 5,6-dimethylbenzimidazole (DMB). Natural seawater was collected from coastal station L4 located in the western English Channel by Dr Glen Wheeler, Marine Biological Association (MBA), UK. Seawater was sterile-filtered directly after collection. Since bacteria are a potential source of B_{12}, cultures were either already axenic (*L. rostrata*), or treated with a cocktail of antibiotics to remove/reduce contaminating bacteria. *P. lutheri*, *T. pseudonana*, *A. carterae, E. gracilis* were grown for 6 days with ampicillin (1 mg/mL), kanamycin (1 mg/mL), and neomycin (0.25 mg/mL). The same cocktail of antibiotics was applied to *O. tauri* for 14-21 days with the addition of streptomycin (50 µg/mL). *C. reinhardtii* and *E. gracilis* were treated with kasaugamycin (75 µg/mL) and ampicillin (50 µg/mL). *A. anophagefferens* cells were exposed sequentially to penicillin (1 mg/mL), neomycin (0.25 mg/mL), streptomycin (0.10 mg/mL), and gentamicin (0.25 mg/mL) treatments, as previously described [S23]. For algal growth assays OD_{730} was measured to quantify growth. Regressions of optical density vs. microscopic cell counts were highly linear (R^2 > 0.97) for species investigated in this study. To monitor bacterial contamination cultures were regularly plated onto LB and TY plates (for freshwater algae) or ASW plates (+0.1% (w/v) glucose, 0.1% (w/v) yeast extract) for marine algae and marine *Synechococcus* strains, or YP plates for *Synechococcus elongatus* PCC7942 and *Synechocystis* sp. PCC6803. For qPCR analysis RNA samples were extracted from cultures of *C. reinhardtii* or *P. tricornutum* grown in TAP or F/2 media respectively in continuous white fluorescent light to mid/late log phase (3-7 days), with and without additions of cobalamin or pseudocobalamin.

Chemicals

Cyanopseudocobalamin was prepared by guided biosynthesis from a culture of *Propionibacterium acidi-propionici* DSM 20273 as described previously S[24], and confirmed by UV-Vis, CD, mass, and NMR spectroscopic analysis [S24].

LC-MS analysis of B_{12} variants

For B_{12} analysis by LC-MS, samples were separated on an Agilent 1100 series HPLC coupled to a Bruker micrOTOF-Q II mass spectrometer using an Ace 5 AQ column (2.1 x 150 mm; Advanced Chromatography Technologies) maintained at 30ºC and with a flow rate of 0.2 mL/min. The mobile phase consisted of 0.1% (v/v) formic acid in water (solvent A) and methanol (solvent B). The initial conditions were set at 95% A, 5% B. The concentration of solvent B was increased with a linear gradient to 70% over 25 min and back to the initial conditions at 30 min. The mass spectrometer was operated in the positive electrospray mode.

Reverse transcription quantitative real-time PCR (RT-qPCR)

Details of the primers and specific amplification conditions used for qPCR are given in Table S3. For each gene, primers were designed for different exons (i.e. they spanned an intron) so that genomic DNA contamination could be assessed. qPCR was carried out using Qiagen SYBR Green MasterMix using 10 ng of cDNA and 0.1 mM of each primer per reaction. Unique single products were verified by melt-curve analysis. No amplification was detected in no-RT control samples, confirming the absence of genomic DNA. Cycle threshold (C_T) values of two technical replicates were calculated using Rotor-Gene Q Software version 2.02, and primer efficiency was calculated with LinRegPCR [S25]. Quantification of each gene of interest was calculated based on the Pfaffl model [S26, S27], using the reference genes: Histone H4, 30S Ribosomal protein S1 (*RPS1*) and TATA box-binding protein (*TBP*) (*P. tricornutum*) [S28], and Actin (*ACT*), Receptor of activated *protein* kinase C 1 (*RACK1*) and Ubiquitin (*UBQ*) for *C. reinhardtii* [S29]. Results were subsequently verified using the relative expression software tool (REST, 2009) [S27].

***C. reinhardtii* reporter lines**

Reporter lines containing the *BLE.GFP* gene driven by the B_{12}-repressible *METE* promoter (Figure 6) were generated in *C. reinhardtii* st.12 background (Table S2) [S30]. To assess the effect of cobalamin versus pseudocobalamin on the *METE* promoter, growth assays were used to test resistance to zeocin in the different treatments. For each transformed line of *C. reinhardtii*, three replicate 'pre-cultures' were set up in 24-well plates on non-selective TAP medium (without zeocin) for each no addition/+Cbl/+PsCbl treatment. After 72 hours, 20 µL of these pre-cultures were inoculated into 2 mL for each treatment, this time with 20 µg/mL zeocin in 24-well plates. The OD_{730} of the culture was determined after 13 days to establish growth of the culture. Where cells had grown this indicated normal expression of the *BLE* gene conferring zeocin resistance. Where cells did not grow, this indicated that the added B_{12} variant had repressed expression of the *BLE* gene.

Supplemental References

S1. Scanlan, D. J, Ostrowski, M., Mazard, S., Dufresne, A., Garczarek, L., Hess, W.R., Post, A.F., Hagemann, M., Paulsen, I. and Partensky, F. (2009). Ecological genomics of marine picocyanobacteria. Microbiol. Mol. Biol. *Rev. 73* , 249–99.

S2. Raux, E., Lanois, A., Levillayer, F., Warren, M.J., Brody, E., Rambach, A. and Thermes, C. (1996). *Salmonella typhimurium* cobalamin (vitamin B_{12}) biosynthetic genes: functional studies in *S. typhimurium* and *Escherichia coli*. J. Bacteriol. *178*, 753–67

S3. Helliwell, K. E., Collins, S., Kazamia, E. K., Purton S. P., Wheeler, G. W., and Smith, A. G. (2014). Fundamental shift in vitamin B_{12} eco-physiology of a model alga demonstrated by experimental evolution. ISME J. *9*, 1446–1455

S4. Schneider, M. J., Ulland, M., and Sloboda, R. D. (2008).A protein methylation pathway in *Chlamydomonas* flagella is active during flagellar resorption. Mol. Biol. Cell. *19*, 4319–27.

S5. Schneider, C. A., Rasband, W. S., and Eliceiri, K. W. (2012). NIH Image to ImageJ: 25 years of image analysis. Nat. Methods *9*, 671–5.

S6. Keeling, P.J., Burki, F., Wilcox, H.M., Allam, B., Allen, E.E., Amaral-Zettler, L.A., Armbrust, E.V., Archibald, J.M., Bharti, A.K., Bell C.J., *et al*. (2014). The marine microbial eukaryote transcriptome sequencing project (MMETSP): illuminating the functional diversity of eukaryotic life in the oceans through transcriptome sequencing. PLoS Biol. *12*, e1001889.

S7. Guillard, R. R. L, and Ryther, J. H. (1962). Studies of marine planktonic diatoms: i. *Cyclotella nana hustedt*, and *Detonula confervacea* (cleve) gran. Can. J. Microbiol. *8*, 229–239.

S8. Guillard, R. R. L (1975) *Culture of Phytoplankton for Feeding Marine Invertebrates.* (Plenum Press, New York, USA) pp 20-25.

S9. Gorman, D. S., and Levine, R. P. (1965). Cytochrome f and plastocyanin: their sequence in the photosynthetic electron transport chain of *Chlamydomonas reinhardi*. Proc. Natl. Acad. Sci. U. S. A. *54*, 1665–9

S10. Guillard, R. R. L., and Hargraves, P. E. (1993). *Stichochrysis immobilis* is a diatom, not a chrysophyte. Phycologia 32, 234–236.

S11. Wilson, W. H., Carr, N. G., and Mann, N. H. (1996). The effect of phosphate status on the kinetics of cyanophage infection in the oceanic cyanobacterium *Synechococcus* sp. WH78031. J. Phycol. 32, 506–516.

S12. Rippka, R., Deruelles, J., Waterbury, J. B., Herdman, M., and Stanier, R. Y. (1979). Generic assignments, strain histories and properties of pure cultures of cyanobacteria. J. Gen. Microbiol. *111*, 1–61.

S13. Altschul, S.F., Madden, T.L., Schäffer, A.A., Zhang, J., Zhang, Z., Miller, W., Lipman, D.J. (1997). Gapped BLAST and PSI-BLAST: a new generation of protein database search programs. Nucleic Acids Res. *25*, 3389–402.

S14. The Uniprot Consortium UniProt: a hub for protein information. (2015). Nucleic Acids Res. *43*, D204–12

S15. Haft D.H., Selengut, J.D., Richter, R.A., Harkins, D., Basu, M.K., and Beck, E. (2013) TIGRFAMs and genome properties in 2013. Nucleic Acids Res. *41*, 387–395

S16. Marchler-Bauer, A., Derbyshire, M.K., Gonzales, N.R., Lu, S., Chitsaz, F., Geer, L.Y., Geer, R.C., He, J., Gwadz, M., Hurwitz, D.I., *et al.* (2014). CDD: NCBI’s conserved domain database. Nucleic Acids Res. *43*, D222–D226.

S17. Finn, R.D., Bateman, A., Clements, J., Coggill, P., Eberhardt, R.Y., Eddy, S.R., Heger, A., Hetherington, K., Holm, L., Mistry, J., *et al.* (2014). Pfam: The protein families database. Nucleic Acids Res. *42*, 222–230.

S18. Galperin, M. Y., Makarova, K. S., Wolf, Y. I., and Koonin, E. V. (2014).Expanded microbial genome coverage and improved protein family annotation in the COG database. Nucleic Acids Res. *43*, D261–9.

S19. Waterhouse, A. M., Procter, J. B., Martin, D. M. A., Clamp, M., and Barton, G. J. (2009). Jalview Version 2--a multiple sequence alignment editor and analysis workbench. Bioinformatics *25*, 1189–91

S20. Finn, R.D., Tate, J., Mistry, J., Coggill, P.C., Sammut, S.J., Hotz, H.R., Ceric, G., Forslund, K., Eddy, S.R., Sonnhammer, E.L., *et al.* (2008). The Pfam protein families database. Nucleic Acids Res. *36*, D281–8.

S21. Tardif, M., Atteia, A., Specht, M., Cogne, G., Rolland, N., Brugière, S., Hippler, M., Ferro, M., Bruley, C., Peltier, G., *et al.* (2012). PredAlgo: a new subcellular localization prediction tool dedicated to green algae. Mol. Biol. Evol. *29*, 3625–39.

S22. Emanuelsson, O., Brunak, S., von Heijne, G., and Nielsen, H. (2007). Locating proteins in the cell using TargetP, SignalP and related tools. Nat. Protoc. *2*, 953–71.

S23. Berg, G. M., Repeta, D. J., and Laroche, J. (2002). Dissolved organic nitrogen hydrolysis rates in axenic cultures of *Aureococcus anophagefferens* (Pelagophyceae): comparison with heterotrophic bacteria. Appl. Environ. Microbiol. *68*, 401–404.

S24. Hoffmann, B., Oberhuber, M., Stupperich, E., Bothe, H., Buckel, W., Konrat, R., and Kräutler, B. (2000). Native corrinoids from *Clostridium cochlearium* are adeninylcobamides: spectroscopic analysis and identification of pseudovitamin B($_{12}$) and factor A. J. Bacteriol. *182*, 4773–82.

S25. Ramakers, C., Ruijter, J. M., Deprez, R. H. L., and Moorman, A. F. M. (2003). Assumption-free analysis of quantitative real-time polymerase chain reaction (PCR) data. Neurosci. Lett. *339*, 62–6.

S26. Vandesompele, J., De Preter, K., Pattyn, F., Poppe, B., Van Roy, N., De Paepe, A., and Speleman, F. (2002). Accurate normalization of real-time quantitative RT-PCR data by geometric averaging of multiple internal control genes. Genome Biol. *3*, RESEARCH0034.

S27. Pfaffl, M. W., Horgan, G. W., and Dempfle, L. (2002). Relative expression software tool (REST) for group-wise comparison and statistical analysis of relative expression results in real-time PCR. Nucleic Acids Res. *30*, e36.

S28. Siaut, M., Heijde, M., Mangogna, M., Montsant, A., Coesel, S., Allen, A., Manfredonia, A., Falciatore, A., and Bowler C. (2007). Molecular toolbox for studying diatom biology in *Phaeodactylum tricornutum*. Gene *406*, 23–35.

S29. Mus, F., Dubini, A., Seibert, M., Posewitz, M. C., and Grossman, A. R. (2007). Anaerobic acclimation in *Chlamydomonas reinhardtii*: anoxic gene expression, hydrogenase induction, and metabolic pathways. J. Biol. Chem. *282*, 25475–86.

S30. Helliwell, K. E., Scaife, M. A., Sasso, S., Araujo, A. P. U., Purton, S., and Smith, A. G (2014). Unraveling vitamin B_{12}-responsive gene regulation in algae. Plant Physiol. *165*, 388–97.
